# No indications for altered EEG oscillatory activity in patients with chronic post-burn itch compared to healthy controls

**DOI:** 10.1038/s41598-022-08742-8

**Published:** 2022-03-25

**Authors:** Samantha K. Millard, Klara Bokelmann, Rik Schalbroeck, Nic J. A. van der Wee, Nancy E. E. van Loey, Antoinette I. M. van Laarhoven

**Affiliations:** 1 Health, Medical, and Neuropsychology Unit, Faculty of Social and Behavioural Sciences, Leiden University, Wassenaarseweg 52, 2333 AK Leiden, The Netherlands; 2 Centre for Pain IMPACT, Neuroscience Research Australia, 139 Barker Street, Randwick, Sydney, NSW 2031 Australia; 3 School of Medical Science, Faculty of Medicine, University of New South Wales, 18 High St, Kensington, Sydney, NSW 2052 Australia; 4 Department of Psychiatry, Leiden University Medical Centre, Albinusdreef 2, 2333 ZA Leiden, The Netherlands; 5 Association of Dutch Burn Centers, Maasstad Hospital, Burn Center, Maasstadweg 21, 3079 DZ Rotterdam, The Netherlands; 6 Department of Clinical Psychology, Utrecht University, Heidelberglaan 1, 3584 CS Utrecht, The Netherlands; 7 Leiden Institute for Brain and Cognition (LIBC), Leiden University, P.O. Box 9600, 2300 RC Leiden, The Netherlands

**Keywords:** Skin diseases, Pain, Pruritus

## Abstract

A large proportion of patients with burn injuries develop chronic itch, which impacts quality of life. The underlying pathophysiological mechanisms are poorly understood. This cross-sectional pilot study investigates whether altered cortical oscillatory processes are involved in chronic post-burn itch. Continuous electroencephalography (EEG) data were recorded during rest and stimulation of non-injured skin, inducing itch (histamine and electrical) and cold-pressor task pain for 15 adults with chronic post-burn itch and 15 matched healthy controls. Quantitative metrics comprised oscillatory power and peak frequencies in theta, alpha, and beta bands. No statistical differences between patients and healthy controls were found in oscillatory activity during rest or stimulation, with Bayesian analysis suggesting equivocal evidence. However, post-traumatic stress symptoms and duration of chronic itch may be associated with changes in oscillatory activity. A lack of differences in cortical oscillatory processing and itch levels at non-injured sites, suggests that itch symptoms have a localised character in this sample of patients with post-burn itch. For future studies, a biopsychological approach with integration of peripheral and central nervous system techniques, linear and non-linear EEG analysis, injured and non-injured stimulation sites, and incorporation of individual characteristics is recommended. Insight into pathophysiological mechanisms underlying chronic post-burn itch could improve diagnostics and treatments.

## Introduction

*Pruritus* (i.e. *itch*) is common following burn injuries: up to 93% of hospital-admitted patients with burn injuries experience acute itch within the first weeks post injury^1–3^. If itch persists after the wound-healing phase of approximately six months, it is termed *chronic post-burn itch*^4^ . Reported prevalence of chronic post-burn itch varies between 70 and 83% at 12 months^1,3^ to 67–73% at 24 months^1,3^. Chronic itch can impact quality of life and daily functioning, as sleep problems and depressive mood are common amongst sufferers^1,5^. Although several factors may increase the likelihood of chronic post-burn itch, including deep dermal injuries (i.e. requiring excision and grafting), larger total body surface area (TBSA) burnt, and post-traumatic stress (PTS) symptoms^1,3,6^, the pathophysiological mechanisms are barely understood^4,6^.

Regarding mechanisms, it is known that histamine levels are elevated inside hypertrophic scars compared to non-injured skin, demonstrating a cutaneous component; however responsiveness to antihistamines is suboptimal^7^. Patients sometimes report neuropathic-like itch sensations (e.g., pins and needles) and, for some, centrally acting agents (e.g., naltrexone, gabapentin, pregabalin) can effectively reduce itch^7–9^, indicating neuropathic components^5,7^. Akin to *chronic pain*^10^, peripheral and central sensitisation processes are assumed to play a role in chronic itch conditions^11–13^, characterised by increased responsiveness of pruriceptive neurons to sub- and suprathreshold itch stimulation. However, when using quantitative sensory testing (QST) as a proxy measure of sensitisation, evidence for central sensitisation in patients with chronic post-burn itch—and other chronic itch conditions (i.e. mainly atopic dermatitis^12^)—is not convincing^14^. The majority of patients with chronic post-burn itch only report itch within burn-scarred areas, and no evidence was found for altered central modulation of itch by itchy or painful stimuli—applied heterotopically—in patients compared to controls^14^. The extent to which neuropathic and central processes are involved in chronic post-burn itch remains unclear (for comprehensive overviews, see^2,4,15–21^).

Relative to chronic itch, chronic pain has been researched far more extensively. Research using electroencephalography (EEG) comparing the neurophysiology of people with and without chronic pain, indicates a major role of altered cortical oscillatory processing in chronic pain^15,16,22,23^. Continuous EEG recordings enable the measurement of electrical brain activity that results from post-synaptic neuronal firing, the frequencies of which have been split into bands that are thought to be functionally or structurally separable^24^. Frequency characteristics, such as power (i.e. signal magnitude) and the peak of different frequencies (i.e. the specific frequency with highest magnitude within a frequency range), are thought to describe neuronal synchrony and, consequently, information flow in the brain^25,26^. Within pain research, patients with chronic pain appear to have lower peak alpha frequency (PAF) at rest than healthy pain-free controls^16,27–29^, although evidence is equivocal^15,30^. This suggests that patients with chronic pain have altered thalamocortical processing that may impact sensory perception, as PAF is thought to reflect information exchange between the thalamus and cortex^31^ or the rate at which we sample sensory information from the environment^32^.

Despite the potential usefulness of studying itch using EEG^33,34^, to date, only one study has investigated neurophysiological processes in patients with chronic post-burn itch^35^. By recording continuous EEG during rest, Miraval and colleagues^35^ suggest that, compared to healthy controls (HCs; *n* = 4 males), patients with chronic post-burn itch (*n* = 4 males) had lower global theta (4–8 Hz) power, both when eyes were open and closed, as well as lower alpha (8–12 Hz) power in occipital areas, and lower beta (12–21 Hz) power in frontal areas when eyes were closed. This provides preliminary evidence for cortical processing differences in patients with chronic post-burn itch compared to HCs, albeit with a small sample size.

The current study aimed to extend this preliminary work by comparing cortical oscillatory activity (i.e. power and peak frequencies) of patients with chronic post-burn itch to HCs during rest as well as during itch and pain stimulations. EEG data were recorded during a cross-sectional study conducted in 2014–2015, which formed the basis of previously published work comparing patients with chronic post-burn itch to HCs on psychophysical measures^14^. In light of continued publication of literature showing altered cortical oscillatory processing in chronic pain patients, as well as Miraval and colleagues’ preliminary study on chronic post-burn itch patients^35^, the current exploratory analysis was formulated.

This investigation advances the currently sparse knowledge of the cortical neurophysiological processes involved in chronic post-burn itch and provides invaluable hypothesis generation for continued research. Findings and further investigations may be useful for assisting diagnosis, establishing optimal treatments, and investigating the efficacy of new treatments in patients suffering from chronic post-burn itch.

## Results

### Participants

From the original sample of 30 participants described by van Laarhoven and colleagues^14^, one healthy female participant was excluded from all analyses due to noisy EEG data. The remaining 15 patients (10 females) and 14 HCs (9 females) were on average 41.6 (± 14.7) and 40.3 (± 13.2) years old, and their numerical rating scale (NRS) baseline levels of itch were 2.8 (± 2.6, range: 0–8) and 0.1 (± 0.3, range: 0–1), respectively. Baseline pain ratings ranged from 0 to 5 for patients, with an average of 1.6 (± 2.0); none of the HCs reported baseline pain. The duration of chronic itch in patients was on average 19.8 years (± 18.0, range: 2.4–64.7), self-reported TBSA affected was 27% (± 19.0, range: 3–68%) and the impact of event (IES) total score, used to indicate level of PTS symptoms, was 23 (± 21.8, range: 0–63). Six patients had clinically relevant levels of PTS symptoms related to their burn injury according to the IES (i.e. score ≥ 26); with an average score of 46.3 (± 10.3, range: 33–63)^36^ . No significant differences in age, sex, or education level were found between patients and HCs.

### Resting state

**Figure 1:**
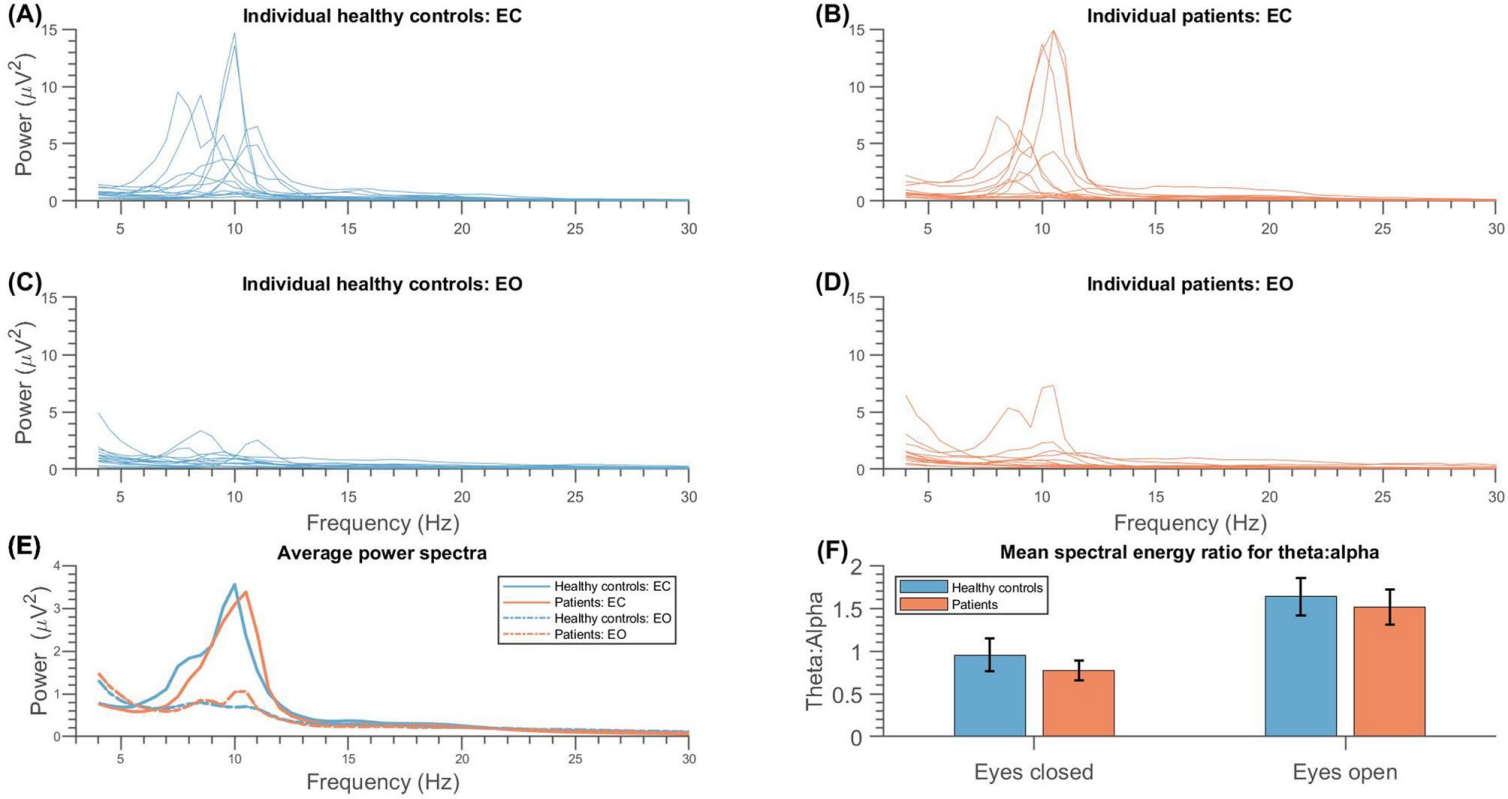
Spectral analysis of resting state activity for patients with chronic post-burn itch (orange) and healthy controls (blue). Spectra of mean power of global resting state activity recorded from all electrodes in (**a**) healthy controls (HCs) with eyes closed, (**b**) patients with eyes closed, (**c**) HCs with eyes open, and (**d**) patients with eyes open. Individual lines represent individual participants for plots (**a–d**). (**e**) Plot of mean power spectra for HCs (blue) and patients (orange), with full lines representing eyes closed (EC) and dashed lines representing eyes open (EO) conditions. Frequency bands of interests are distinguished left to right as white (theta: 4–7.5 Hz), grey (alpha: 8–12.5 Hz), and white (13–30 Hz) panels. (**f**) Mean spectral energy ratio for theta:alpha. With eyes closed, there is less theta compared to alpha power, whereas with eyes open there is more theta compared to alpha power, for both patients (orange) and HCs (blue). The theta:alpha between the patients (EC: Mean (M) = .77, standard deviation (SD) = .45, n = 14; EO: M = 1.51, SD = .74, n = 13) and the HCs (EC: M = .95, SD = .72, n = 14; EO: M = 1.64, SD = .82, n = 14) was not statistically significant for EC (t(26) = .81, p = .428) or EO (t(25) = .41, *p* = .680).

**Figure 2:**
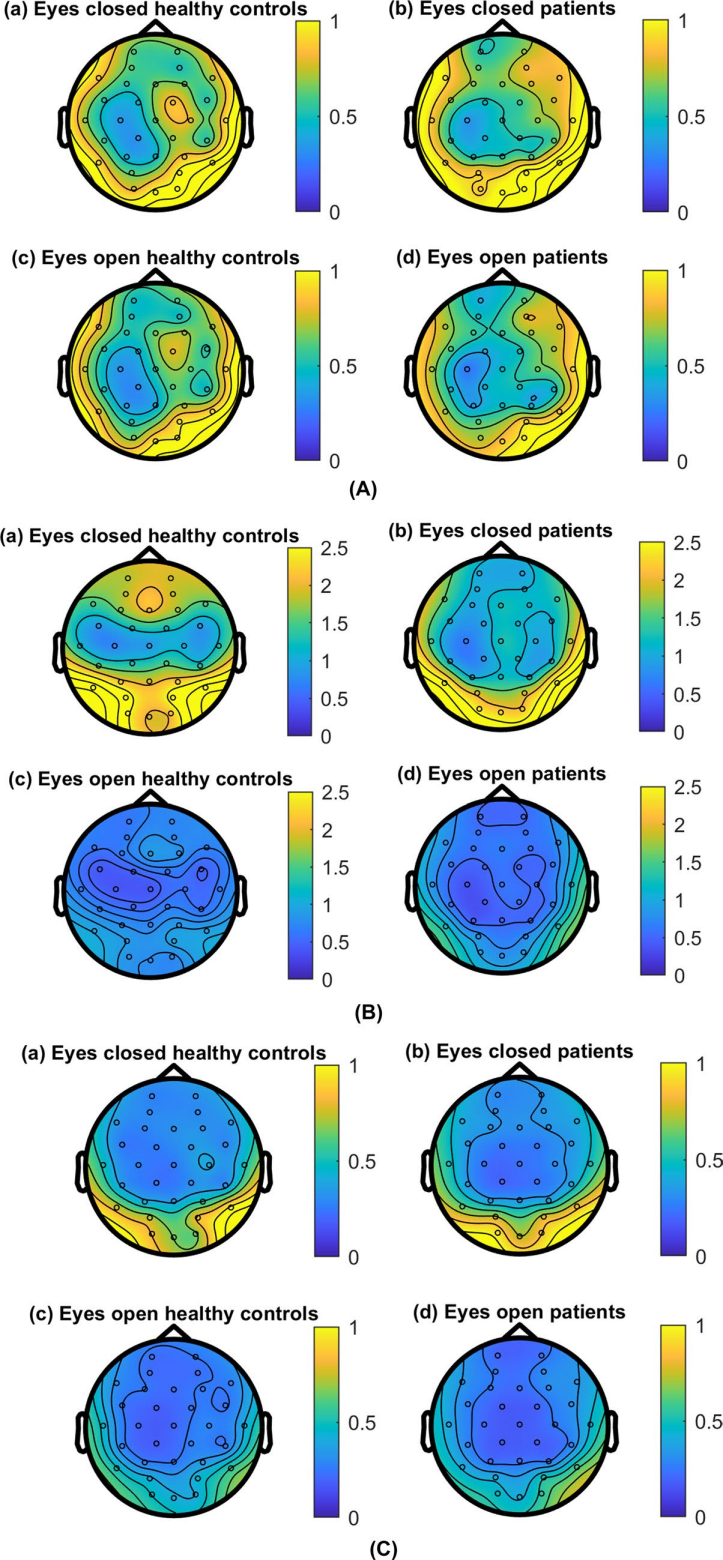
Topoplots visualising oscillatory power during resting state. (**A**) Topography of resting state theta (4–7.5 Hz) power (µV^2^) for patients with chronic post-burn itch and healthy controls. Averaged for healthy control participants during (**A.a**) eyes closed and (**A.c**) eyes open, and for chronic post-burn itch patients during (**A.b**) eyes closed and (**A.d**) eyes open. (**B**) Topography of resting state alpha (8–12.5 Hz) power (µV^2^) for patients with chronic post-burn itch and healthy controls. Averaged for healthy control participants during (**B.a**) eyes closed and (**B.c**) eyes open, and for chronic post-burn itch patients during (**B.b**) eyes closed and (**B.d**) eyes open. (**C**) Topography of resting state beta (13–30 Hz) power (µV^2^) for patients with chronic post-burn itch and healthy controls. Averaged for healthy control participants during (**C.a**) eyes closed and (**C.c**) eyes open, and for chronic post-burn itch patients during (**C.b**) eyes closed and (**C.d**) eyes open.

Brain oscillations during resting state are visualised in topographical plots for theta, alpha, and beta power (see Fig. 2a–c, respectively), which do not indicate broad differences between the patients and controls. This is confirmed by the RM-ANOVAs on the global data (i.e. all EEG electrodes) showing no significant main effects of group (i.e. patient/HC) on power or mean peak frequency (see also Supplementary Information A) in any band (*p* ≥ 0.350), with Bayesian analysis suggesting there was inconclusive evidence for both the null and alternative hypotheses (BF_10_ = 0.38–0.63; Table 1). However, a significant main effect of condition (i.e. eyes closed [EC] / eyes open [EO]) on power was found in the theta (*F*(1, 25) = 9.21, *p* = 0.006, *η*_*G*_^*2*^ = 0.04, BF_10_ = 3.30) and alpha bands (*F*(1, 25) = 34.97, *p* < 0.001, *η*_*G*_^*2*^ = 0.19, BF_10_ = 215.05), with Bayesian analysis suggesting positive and very strong evidence towards these alternative hypotheses, respectively. Additionally, significant main effects of condition on mean peak frequencies were found in the theta and beta bands with very strong and strong evidence respectively (Theta: *F*(1, 25) = 24.51, *p* < 0.001, *η*_*G*_^*2*^ = 0.06, BF_10_ = 506.02; Beta: *F*(1, 25) = 17.13, *p* < 0.001, *η*_*G*_^*2*^ = 0.14, BF_10_ = 101.59). Theta and alpha power, as well as theta and beta mean peak frequencies, were significantly higher during EC (Fig. 1a, b, e) than EO (Fig. 1c–e) across all participants (also see Supplementary Information B).

**Table 1:** Comparison of global power and mean peak frequency outcomes between patients (*n* = 13) and healthy controls (HC; *n* = 14) during eyes closed (EC) and eyes open (EO) resting states.

Frequency band	Mean (SD) patients		Mean (SD) HCs		Condition (EC/EO)				Group (patient/HC)				Condition * group			
	EC	EO	EC	EO	F (1,25)	*p*-value	ηG^2^	BF_10_	F (1,25)	*p*-value	ηG^2^	BF_10_	F (1,25)	*p*-value	ηG^2^	BF_10_
**Power**																
Theta﻿	.67 (.58)	.51 (.36)	.84 (.74)	.55 (.27)	9.21	.006**	.04	3.30	.38	.544	.01	.46	.45	.508	< .01	.41
Alph﻿﻿a	1.95 (2.31)	.73 (.93)	1.81 (1.50)	.59 (.39)	34.97	< .001***	.19	215.05	.01	.939	< .01	.38	< .01	.964	< .01	.36
Beta	.20 (.17)	.18 (.14)	.20 (.12)	.18 (.11)	1.98	.172	< .01	.62	< .01	.949	< .01	.63	.11	.748	< .01	.39
**Mean peak**																
Theta	5.67 (.35)	5.50 (.31)	5.79 (.56)	5.56 (.42)	24.51	< .001***	.06	506.02	.21	.651	.01	.55	.21	.652	< .01	.39
Alpha	10.04 (.57)	10.01 (.32)	9.81 (.56)	9.96 (.52)	.96	.338	.01	.41	.45	.509	.01	.52	.73	.402	.01	.47
Beta	17.19 (1.00)	17.99 (.98)	16.85 (.92)	17.62 (1.22)	17.13	< .001***	.14	101.59	.91	.350	.03	.50	.03	.876	< .01	.35

The displayed means are based on the untransformed variables. For theta and alpha power, ﻿the analysis of variance was conducted using square-root transformed variables. SD = standard deviation. *η*_*G*_^*2*^ = generalised eta squared. BF_10_ = Bayes factor, evidence towards alternative hypothesis.

*p < .05; **p < .01; ***p < .001.

Figure 1f shows the theta:alpha ratios of patients and HCs. The t-tests did not suggest significant differences in theta:alpha ratios between patients (*EC:* 0.77 ± 0.43; *EO:* 1.51 ± 0.74) and HCs (*EC:* 0.95 ± 0.72; *EO:* 1.64 ± 0.82) for EC (*t*(26) = 0.81, *p* = 0.428) or EO conditions (*t*(25) = 0.41, *p* = 0.689).

As with globally, theta, alpha, and beta activity in the four ROIs appear to be comparable between the groups according to the topoplots (Fig. 2a–c), with the exception of alpha power in the frontal regions that seems lower in the patients than in the HC during the eyes closed resting state. However, no significant interactions or main effects of group were found for any of the frequency bands in our pre-defined regions of interest (ROIs) (Supplementary Information C). Again, significant main effects of condition (i.e. EO/EC) were found for power in several ROIs in the theta and beta bands (Table S-C.1), as well as for mean peaks in most ROIs across all three frequency bands (Table S-C.2).

### Stimulation

#### Histamine itch

Although the sample size decreased to enable EEG analysis, akin to previously published behavioural results^14^, differences in average histamine itch ratings between the patients (*n* = *10,* 3.1 ± 1.6) and HCs (*n* = *12,* 2.8 ± 2.1; *F*(1,20) = 0.10, *p* = 0.757, *η*_*p*_^2^ = 0.01) whose EEG data were assessed for this outcome were nonsignificant. Two independent-samples t-tests showed no differences in power or mean peak frequencies between groups in the theta, alpha, or beta bands (*p* ≥ 0.396), with Bayesian statistics suggesting that evidence was inconclusive (BF_10_ = 0.39–0.51; Table 2A). Exploratory RM-ANOVAs for histamine are displayed in Supplementary Information D; Tables S-D.1 and S-D.3 show that no effects of group on power or mean peak frequencies were found.

**Table 2:** Comparisons of global power outcomes (A) and mean peak frequency outcomes during histamine stimulation, (B) during electrical “must-scratch” threshold stimulation, and (C) during painful cold pressor task (CPT) stimulation between patients and healthy controls (HCs).

t-test							
Frequency band	Mean (SD) patients (*n* = 10)	Mean (SD) HCs (*n* = 12)	Mean difference (SE)	*t*-value (df = 20)	*p*-value	BF_10_	
**(A)**							
Power	Theta	.49 (.23)	.55 (.24)	.06 (.10)	.63	.534	.45
	Alpha	.37 (.30)	.46 (.38)	.08 (.15)	.56	.581	.43
	Beta	.19 (.14)	.19 (.13)	< .01 (.06)	.08	.937	.39
Mean peak	Theta	5.42 (.20)	5.53 (.36)	.11 (.13)	.87	.396	.51
	Alpha	9.89 (.27)	9.79 (.33)	−.09 (.13)	−.73	.475	.47
	Beta	18.28 (1.02)	18.18 (1.04)	−.10 (.44)	−.23	.822	.39

t-test							
Frequency band	Mean (SD) patients (*n* = 13)	Mean (SD) HCs (*n* = 13)	Mean difference (SE)	*t*-value (df = 24)	*p*-value	BF_10_	
**(B)**							
Theta	.57 (.49)	.53 (.35)	-.01 (.10)	-.10	.923	.36	
Beta	.19 (.11)	.17 (.13)	-.03 (.05)	-.53	.606	.40	

Mann–Whitney U test							
	Median (IQR) patients (*n* = 13)	Median (IQR) HCs (*n* = 13)		*U*	*p*-value	BF_10_	
Alpha	.35 (.63)	.32 (.79)		84	1.00	.37	

t-test							
Frequency band	Mean (SD) patients (*n* = 11)	Mean (SD) HCs (*n* = 12)	Mean difference (SE)	*t*-value (df = 21)	*p*-value	BF_10_	
**(C)**							
Theta	.47 (.17)	.45 (.21)	−.02 (.05)	−.29	.773	.39	
Alpha	.37 (.27)	.40 (.27)	.03 (.08)	.37	.717	.40	
Beta	.22 (.15)	.19 (.14)	−.03 (.06)	−.56	.583	.42	

The displayed means are based on the untransformed variables. For the variables, in panel A, beta power, beta mean peak; in panel B, theta and beta power, and in panel C, all displayed frequency bands the t-tests were conducted using square-root transformed variables. SD = standard deviation. SE = standard error. df = degrees of freedom. BF_10_ = Bayes factor, evidence towards alternative hypothesis. IQR = inter-quartile range.

#### Electrical “must-scratch” threshold

As in van Laarhoven and colleagues^14^, differences in average electrical itch ratings between patients (*n* = *13,* 4.4 ± 2.9) and HCs (*n* = *13,* 3.5 ± 2.3; *F*(1,24) = 0.73, *p* = 0.401, *η*_*p*_^2^ = 0.03) were also nonsignificant for those participants included in EEG analysis. Independent-samples t-tests did not show significant differences between groups for theta and beta bands (*p* ≥ 0.606); neither did the Mann–Whitney U test for alpha power (*p* = 1.00), with Bayesian statistics suggesting that evidence was inconclusive (BF_10_ = 0.36-0.40; Table 2B). Supplementary Information E includes exploratory RM-ANOVAs for power during electrical stimulation; no effects of group on power were found but main effects of stimulation side and ROI are displayed.

#### Cold presser task (CPT) pain

As previously^14^, differences in average CPT pain ratings between patients (*n* = 11*,* 3.8 ± 3.0) and HCs (*n* = *12,* 3.1 ± 2.6; *F*(1,21) = 0.43, *p* = 0.520, *η*_*p*_^2^ = 0.02) were nonsignificant. An independent samples t-test did not show any significant differences in power between groups for the theta, alpha, or beta bands (*p* ≥ 0.583), with Bayesian statistics suggesting that evidence was inconclusive (BF_10_ = 0.39–0.42; Table 2C). Exploratory RM-ANOVAs for the CPT are displayed in Supplementary Information F; Table S-F.1 shows that no effects of group on power were found.

### Correlations

Correlations were conducted between several individual characteristics (i.e. duration of chronic itch, levels of PTS symptoms, TBSA, age, baseline pain, and baseline itch) and oscillatory outcomes.

Correlation coefficients between power during resting state and duration of chronic itch in patients are displayed in Table 3. There were strong negative correlations for EC and EO theta, alpha, and beta power (*r*_*S*_ < −0.67, *p* < 0.013 for all tests, see Table 3), suggesting that those who have experienced chronic itch for longer have lower resting state power in these bands. Correlations between mean peak frequency and duration of chronic itch were only significant for the theta band (*r*_*S*_ < −0.63, *p* < 0.016 for both tests, see Table 3); lower mean peak theta frequency during EC and EO were, respectively, moderately and strongly associated with longer duration of chronic itch.

**Table 3:** Spearman's correlation coefficient (*r*_*s*_) between power and mean peak frequencies during rest, with eyes closed (EC) or eyes open (EO), and stimulation states in theta, alpha, and beta bands with patients’ duration of chronic itch experience.

		*n*	Theta		Alpha		Beta	
			*r*_*s*_	*p*-value	*r*_*s*_	*p*-value	*r*_*s*_	*p*-value
**Resting state**								
Power	EC	14	−.76	.002**	−.73	.003**	−.77	.001**
	EO	13	−.67	.013*	−.79	.001**	−.69	.010*
Mean Peak	EC	14	−.63	.016*	.21	.469	.13	.658
	EO	13	−.81	.001**	.22	.481	−.17	.577
**Histamine stimulation**								
Power		10	−.46	.185	−.59	.073	−.69	.028*
Mean Peak		10	−.62	.056	.18	.614	.47	.166
**Electrical stimulation**								
Power		13	−.45	.127	−.74	.004**	−.74	.004**
**CPT stimulation**								
Power		11	−.18	.601	−.57	.070	−.64	.035*

*p < .05; **p < .01; ***p < .001.

During histamine stimulation, lower beta band power was moderately correlated with longer duration of chronic itch (*r*_*S*_ = −0.69, *p* = 0.028). Duration of chronic itch had strong negative correlations with alpha and beta power during electrical stimulation (*r*_*S*_ = −0.74, *p* = 0.004 for both tests, see Table 3) and a moderate negative correlation with beta power during CPT stimulation (*r*_*S*_ < -0.64, p < 0.035).

Correlation coefficients between power and total IES scores (i.e. reflecting PTS symptoms) were nonsignificant during resting state and electrical stimulation (*p* ≥ 0.085 in all tests, see Table 4). During the remaining stimulation states, there were strong negative correlations between theta power and total IES scores (*r*_*S*_ < -0.70 *p* < 0.016, see Table 4). There were moderate or strong negative correlations with IES for alpha and beta power during histamine stimulation (*r*_*S*_ < −0.71, *p* < 0.021, see Table 4) and for alpha power during CPT stimulation (*r*_*S*_ = *−*0.68* p* = 0.022). This indicates that higher levels of PTS symptoms were associated with lower theta power during itch and pain stimulations, as well as lower alpha and beta power during itch stimulations. Correlations between mean peak frequencies and IES outcomes were nonsignificant during resting state and during histamine stimulation (*p* ≥ 0.061 in all tests, see Table 4). Duration of chronic itch and level of PTS symptoms were not significantly correlated (*r*_*S*_ = −0.05, *p* = 0.864).

**Table 4:** Spearman's correlation coefficient (*r*_*s*_) between power and mean peak frequencies during rest, with eyes closed (EC) or eyes open (EO), and stimulation states in theta, alpha, and beta frequency bands with patients’ score on the impact of event scale measuring post-traumatic stress symptoms.

	*n*		Theta		Alpha		Beta	
			*r*_*s*_	*p*-value	*r*_*s*_	*p*-value	*r*_*s*_	*p*-value
**Resting state**								
Power	EC	14	−.24	.411	.08	.793	−.42	.133
	EO	13	−.48	.096	−.07	.830	−.48	.096
Mean Peak	EC	14	.20	.486	−.29	.315	−.13	.061
	EO	13	−.15	.627	.23	.457	.66	.844
**Histamine stimulation**								
Power		10	−.75	.012*	−.71	.021*	−.76	.010*
Mean Peak		10	−.45	.197	−.07	.854	.17	.649
**Electrical stimulation**								
Power		13	−.50	.085	−.22	.475	−.27	.372
**CPT stimulation**								
Power		11	−.70	.016*	−.68	.022*	−.45	.163

*p < .05; **p < .01; ***p < .001.

The weak to moderate correlations between TBSA and power or mean peak in any of the frequency bands were not statistically significant during resting state or stimulations (*p* ≥ 0.058, see Table 5), with the exception of alpha and beta power during histamine stimulation (*r*_*S*_ < -0.69, *p* < 0.026, see Table 5).

**Table 5:** Spearman's correlation coefficient (*r*_*s*_) between power and mean peak frequencies during rest, with eyes closed (EC) or eyes open (EO), and stimulation states in theta, alpha, and beta frequency bands with patients’ total injured body surface area.

		*n*	Theta		Alpha		Beta	
			*r*_*s*_	*p*-value	*r*_*s*_	*p*-value	*r*_*s*_	*p*-value
**Resting state**								
Power	EC	14	−.47	.088	−.28	.335	−.50	.068
	EO	13	−.49	.092	−.28	.360	−.40	.174
Mean Peak	EC	14	−.21	.462	.16	.597	−.11	.707
	EO	13	−.19	.526	.18	.550	.08	.808
**Histamine stimulation**								
Power		10	.60	.070	−.69	.026*	−.74	.014*
Mean Peak		10	.45	.194	−.20	.575	.31	.379
**Electrical stimulation**								
Power		13	−.51	.076	−.34	.251	−.42	.156
**CPT stimulation**								
Power		11	−.59	.058	−.50	.121	−.35	.293

*p < .05; **p < .01; ***p < .001.

Post-hoc analyses to explore the role of age in neurophysiological processing obtained significant correlations between age and theta and beta power during EO resting state (*r*_*S*_ < *−*0.63, *p* < 0.022, see Table 6A) as well as beta mean peak during both EC and EO resting states in patients (*r*_*S*_ > 0.59, *p* < 0.035, see Table 6A). Furthermore, theta and alpha power was significantly negatively related to patients’ age in all three stimulation states (*r*_*S*_ < *−*0.63, *p* < 0.021, see Table 6A), and beta mean peak during histamine stimulation was positively correlated with age (*r*_*S*_ = 0.76, *p* = 0.011, see Table 6A). There was no significant association between age and the duration of chronic post-burn itch (*r*_*s*_ = 0.03, *p* = 0.930) within the patient group. In healthy controls only one significant correlation with age was found, namely between older age and lower alpha mean peak during EC resting state (*r*_*S*_ = *−0.60*, *p* = 0.025, see Table 6B).

**Table 6:** Spearman's correlation coefficient (*r*_*s*_) between (A) patients’ (B) healthy controls’ age and power and mean peak frequencies during rest, with eyes closed (EC) or eyes open (EO), and stimulation states in theta, alpha, and beta bands.

		*n*	Theta		Alpha		Beta	
			*r*_*s*_	*p*-value	*r*_*s*_	*p*-value	*r*_*s*_	*p*-value
**(A)**								
**Resting state**								
Power	EC	14	−.39	.175	−.15	.615	−.41	.144
	EO	13	−.71	.007**	−.41	.168	−.63	.022*
Mean Peak	EC	14	−.002	.994	−.49	.078	.61	.021*
	EO	13	−.14	.655	−.30	.316	.59	.035*
**Histamine stimulation**								
Power		10	−.87	.001**	−.83	.003**	−.62	.054
Mean Peak		10	−.22	.533	−.47	.174	.76	.011*
**Electrical stimulation**								
Power		13	−.76	.002**	−.63	.021*	−.48	.098
**CPT stimulation**								
Power		11	−.88	< .001***	−.73	.011*	−.53	.096
**(B)**								
**Resting state**								
Power	EC	14	−.14	.637	−.36	.208	−.23	.436
	EO	14	−.05	.876	−.07	.817	.06	.852
Mean Peak	EC	14	.04	.887	−.60	.025*	.08	.782
	EO	14	.04	.887	−.31	.281	−.07	.805
**Histamine stimulation**								
Power		10	−.43	.167	−.31	.331	−.20	.542
Mean Peak		10	.03	.931	−.41	.183	−.25	.430
**Electrical stimulation**								
Power		13	−.29	.334	−.24	.426	−.13	.681
**CPT stimulation**								
Power		12	−.55	.067	−.23	.471	−.43	.159

*p < .05; **p < .01; ***p < .001.

Six patients reported baseline pain at the start of the experiment. Correlations were calculated including all patients except for two who reported pain unrelated to their burn injury. The correlations of baseline pain with power and mean peaks were nonsignificant across resting and stimulation states for all three frequency bands (*p* ≥ 0.085 for all tests, see Table 7A). Similarly, correlations of baseline itch in patients with power and mean peaks were nonsignificant for all frequency bands (*p* ≥ 0.053 for all tests, see Table 7B).

**Table 7:** Spearman's correlation coefficient (*r*_*s*_) between power and mean peak frequencies during rest, with eyes closed (EC) or eyes open (EO), and stimulation states in theta, alpha, and beta frequency bands with baseline (A) pain ratings. (B) itch ratings in burn-injured patients. Individuals with pain unrelated to burns were excluded.

		*n*	Theta		Alpha		Beta	
			*r*_*s*_	*p*-value	*r*_*s*_	*p*-value	*r*_*s*_	*p*-value
**(A)**								
**Resting state**								
Power	EC	12	.27	.396	.08	.817	.14	.667
	EO	11	−.16	.640	.12	.716	−.11	.738
Mean Peak	EC	12	.36	.256	.22	.490	−.01	.972
	EO	11	.39	.239	−.50	.120	.54	.085
**Histamine stimulation**								
Power		10	−.44	.203	−.28	.441	< .01	1.00
Mean Peak		10	−.06	.880	−.08	.821	.55	.099
**Electrical stimulation**								
Power		12	−.15	.650	.05	.880	.25	.431
**CPT stimulation**								
Power		11	−.43	.188	−.26	.433	.05	.884
**(B)**								
**Resting state**								
Power	EC	14	.20	.493	−.10	.739	.14	.644
	EO	13	.08	.793	−.01	.978	.06	.835
Mean Peak	EC	14	.11	.711	.31	.286	.10	.734
	EO	13	.24	.431	−.41	.165	.46	.115
**Histamine stimulation**								
Power		10	.25	.490	.34	.335	.30	.404
Mean Peak		10	−.30	.394	.63	.053	−.16	.669
**Electrical stimulation**								
Power		13	.32	.290	.48	.095	.32	.286
**CPT stimulation**								
Power		11	.23	.503	.38	.244	.23	.503

## Discussion

The present results suggest a lack of differences in oscillatory activity between patients with chronic post-burn itch and matched HCs; however, within patients some associations were found between oscillatory activity and patient characteristics. When comparing patients to HCs, no significant differences were found in resting state global power with eyes open or eyes closed. Equivocal Bayes factors support these results. Further exploratory analyses found lateralisation and ROI effects irrespective of group, but no differences in the power or peak frequencies of theta, alpha, or beta frequency bands between patients and HCs during rest or during stimulation of non-injured skin with histamine-induced itch, electrically-induced itch, or CPT-induced pain. A lack of group differences in oscillatory measures between patients and HCs indicates that the mechanisms involved in chronic post-burn itch may be primarily peripheral rather than related to altered central cortical processing. However, exploratory correlations within patients suggest that broad comparisons between patients and HCs may be too simplistic, as differences may change with longer chronic itch experience and higher levels of PTS symptoms.

The lack of group differences in the present findings contradict those of Miraval and colleagues^35^, who found that during rest, compared to HCs, patients with chronic post-burn itch had higher global theta power, both when eyes were open and closed, as well as lower alpha power in occipital areas and lower beta power in frontal areas when eyes were closed. The authors^35^ clearly state that they conducted a preliminary study, as they only tested four patients. They^35^ also reported that significant differences in oscillatory activity between patients and HCs disappeared when the patient who had been experiencing chronic itch for 50 years was removed; the three other patients had been experiencing chronic itch for up to three years.

Present exploratory correlations within patients suggest that alterations in cortical oscillations might change the longer a patient has had chronic post-burn itch. If chronic itch duration is indeed a key factor in oscillatory alterations, this may explain why broad differences in the oscillatory activity of patients and HCs were not found in the present study, as the duration for which the present sample of 15 patients had been living with chronic itch varies from 2.4 to 64.7 years. This, and Miraval and colleagues’^35^ divergent findings due to removal of one patient, indicates that broad comparisons between patients and HCs may be too simplistic. Although the lack of significant associations between the healthy individual’s age and most of the EEG parameters (except for peak alpha frequency) does not point in this direction, it cannot be ruled out that the associations between altered cortical processes and disease duration may partly be related to ageing, as previous research indicates neurophysiological changes related to ageing^37,38^. Larger sample sizes are required to disentangle the effects of ageing and chronic itch duration on oscillatory outcomes.

Higher levels of PTS symptoms may also be associated with changes in several frequency characteristics, both during rest and stimulation. Duration of chronic itch and PTS symptoms themselves were not associated in the present sample, so their associations are likely to be independent of each other. Levels of PTS symptoms have been linked to increased likelihood of continued itch in previous research^3,39^. Therefore, although exploratory, our present findings suggest that sensory processing is altered at the cortical level in patients with chronic post-burn itch with more PTS symptoms. Future investigations into the potentially progressive nature of cortical alterations in oscillatory activity and the impact of PTS in patients with chronic post-burn itch are warranted. Note however, that despite a strong correlation with PTS symptoms, the IES only assessed two out of four symptom clusters of PTS^40,41^. More in-depth assessments may be required in future, for instance using causal longitudinal designs or experimental designs comparing patients with and without PTS symptoms.

Comparing to the broader literature, we found expected standard differences between eyes open and eyes closed resting state oscillatory activity^42^, with Bayesian analysis suggesting there was positive and strong evidence for this. Similar to a handful of chronic pain papers, the present study found no differences in PAF between patients and HCs^15,30^. However, present findings differ from the majority of chronic pain research, as the present study found neither lower PAF in patients compared to HCs^16,27–29^, nor various power alterations at rest^43–46^ and during pain stimulation^47^. Thus, although both acute itch and pain are thought to have considerable overlap centrally (e.g. both involve motor, somatosensory, and limbic areas, as well as the thalamus in particular^20,21^), the chronic states of these perceptually distinct sensations should not be considered analogous.

The nuances within and between chronic itch conditions should also be considered. For example, other research shows a subgroup of patients with chronic post-burn itch are responsive to centrally acting agents, suggesting that central nervous system components play an important role^7–9^. However, another major indicator of cortical alterations and central sensitisation would be increased itch sensation in non-injured skin^10,11^. In this sample of patients, most only reported itch within burned areas, and responses to stimulation of non-injured skin did not differ substantially between patients and HCs^14^. Therefore, chronic post-burn itch, at least in this sample, appears to have a localised nature. Itch is highly prevalent in those with burn injuries, particularly during the wound healing phase, and scar tissue can remain for the rest of their lives^1,3,6^, which can produce continued peripheral input. Neurophysiological oscillatory findings in other populations with chronic itch may be different from those with chronic post-burn itch, although the psychophysical evidence for central sensitisation in other chronic itch conditions—mainly atopic dermatitis—is also limited^12^. To disentangle peripheral and central factors, it would be valuable to follow patients from the acute to chronic phase after burn injuries to assess potential alterations in neurophysiological processing related to injured and non-injured sites during this transition.

We will highlight three assumptions made that should be considered when interpreting our findings. Firstly, comparing patients to HCs assumes that the patient group was sufficiently homogeneous in terms of the duration of chronic itch, clinical itch levels, as well as extent, cause, and severity of burn injuries. Rather than this simple comparison, future research could use the present exploratory findings as hypothesis-generation: irrespective of the effects of ageing, researchers should determine whether duration of chronic itch or levels of PTS symptoms play a role in altered oscillatory activity for those with chronic post-burn itch. Such research could, for example, produce the recommendation that burn-injured patients be screened for PTS symptoms in order to provide appropriate support and potentially prevent development of itch-related cortical alterations and chronic itch. Secondly, some individual characteristics used for correlations in this study were self-reported by patients and should thus be interpreted with a degree of caution. The TBSA, for example, is a measure normally assessed by doctors at the time of injury, however, it is possible that some patients may recall their TBSA incorrectly if it has been many years since their injury. Lastly, using the Fourier transform assumes that EEG oscillations can be decomposed into sinusoidal signals, which could overlook critical features of EEG signal structure. Conducting additional non-linear analyses, such as multiscale entropy analyses^48^, could be of added value to further elucidate the mechanisms underlying chronic post-burn itch.

We will also highlight three methodological limitations and their associated future recommendations. Firstly, 50% of the data collected during short electrical itch stimulations was lost during EEG preprocessing due to noisy data (i.e. muscle artefacts), producing insufficient data to examine how oscillatory activity was altered during an itch modulation procedure, for which the behavioural outcomes were previously published^14^. Future research should increase the number of stimulations obtained if possible. Secondly, due to the low spatial resolution of the 32-channel EEG set-up used, we were unable to conduct source localisation analysis. Use of a high-density EEG set-up, preferably in combination with MRI^49^ is recommended to further clarify underlying processes. Lastly, the present study used pre-defined ROIs that were unable to identify potential differences between patients and controls that were indicated by topographical plots. Future research could investigate potential differences in frontal alpha between patients and controls at rest, by using the present topographical plot to define ROIs or by using cluster-based analyses for further exploration.

To conclude, this is the first study to compare power and peak oscillatory frequencies in patients with chronic post-burn itch and matched healthy controls using continuous EEG during both rest and stimulation. Exploratory frequentist analyses showed no broad significant differences in oscillatory activity between patients and healthy controls, with Bayesian analyses suggesting that evidence was equivocal. When integrating the present findings with the existing evidence (i.e., no clear enhanced itch sensitivity outside the injured areas, spontaneous itch localised around the burn affected areas, and generally suboptimal response to systemic antihistamines), we can now cautiously hypothesise that the mechanisms involved in chronic post-burn itch are not primarily centrally driven. Future research is recommended to place greater emphasis on communication between the peripheral and central nervous system at different levels, as well as the involvement of individual burn-related and psychological characteristics (e.g. PTS symptoms and duration of chronic itch), and complementary analyses of the EEG signals that do not follow linear patterns. Continued investigation into the mechanisms involved in chronic post-burn itch compared to chronic pain or more wide-spread itch conditions (e.g. due to skin or systemic conditions) would further disentangle the potentially divergent mechanisms at play.

## Methods

EEG-related methods (see^14^ for full experimental details) follow guidelines for reporting magneto-/electro-encephalography (MEEG) data set by the Committee on Best Practice in Data Analysis and Sharing (COBIDAS)^50^.

This study was conducted according to the Declaration of Helsinki. The Medical Ethics Review Committee Regio Arnhem-Nijmegen approved the protocol (NL43955.091.13). Leiden University Medical Centre (LUMC) gave permission to conduct experimentation at LUMC (Department of Psychiatry).

### Participants

Fifteen patients with chronic post-burn itch and 15 sex- and age-matched HCs comprised the sample^14^. Individuals were aged 18 or over and had sufficient understanding of Dutch. Additional criteria for patients were spontaneous itch for at least 6 months after burn injury^3,4^ and appropriate unaffected skin to apply somatosensory stimulations. Exclusion criteria included: chronic itch or pain unrelated to burns, multiple sclerosis, diabetes, psychotic disorders or other psychopathology unrelated to burns, pacemakers, epilepsy, claustrophobia, diagnosed histamine hypersensitivity, pregnancy, colour-blindness, or extensive face or head injuries that might interfere with EEG measurements. One patient with Raynaud’s phenomenon abstained from the cold pressor task (CPT). After receiving written information, interested participants were telephone-screened; a medical doctor was consulted for doubts regarding exclusion^14^. All participants were 18 years or older and provided informed consent themselves.

### Measures

Measures of TBSA affected, itch duration, and PTS symptoms were used in the current analyses. Patients were asked what percentage of their TBSA was affected by burns, and how many years ago the burn incident took place (see^14^ for full details). Patients’ PTS symptoms were measured with the validated Dutch version of the Impact of Event Scale (IES; Cronbach’s alpha 0.94, a 15-item self-report measure (total scores ranging from 0 to 75) used to assess the intrusive and avoidant symptom clusters of PTS^40,41^. In adult patients with burns, the IES was demonstrated to be a good indicator of PTS^51^. Scores ≥ 26 indicated clinically significant PTS symptoms^36^.

### EEG recording

EEG data were recorded continuously in an unshielded room using a 32-channel ActiveTwo BioSemi system (BioSemi, Amsterdam), with driven right leg (DRL) as ground and common mode sense (CMS) as online reference. Data were sampled at 1024 Hz, band-pass filtered (0.1–100 Hz), and digitised with a 24-bit analogue-to-digital converter. Electrodes (Ag–AgCl) were positioned according to the international 10–20 system (locations: Fp1, AF3, F7, F3, FC1, FC5, T7, C3, CP1, CP5, P7, P3, Pz, PO3, O1, Oz, O2, PO4, P4, P8, CP6, CP2, C4, T8, FC6, FC2, F4, F8, AF4, Fp2, Fz, and Cz). Impedances were primarily between − 25 and + 25 kOhms. External electrodes were applied above and below the eyes, the outer canthi of each eye, and the left and right mastoids.

### Procedure

#### Resting-state recordings

Each participant attended a two hour experimental session after providing written informed consent. Comfortably seated, resting state EEG data were recorded for 3 min with eyes closed (EC) and eyes open (EO) at the beginning (T1) and the end (T2) of the experiment. Participants were instructed to relax but stay awake during EC, and to relax and concentrate on a point opposite them during EO.

#### Stimulation-state recordings

EEG data were recorded during application of mechanical, electrical, histamine, and CPT stimuli (see^14^ for full details). Due to imprecise application timing, signal during mechanical stimuli was not analysed. An electrical itch procedure established individual “must-scratch” thresholds^52^ . Histamine (0.6% as diphosphate-monohydrate)^53^ was applied by iontophoresis for 2.5 min to induce itch. During itch stimulations, participants were instructed not to scratch but were given breaks and additional rest on request. The water for the painful CPT was made at circa 4 °C, in which participants submerged their hand for a maximum of 1 min. During stimulations, participants’ itch or pain sensitivity were measured by NRSs, ranging from 0 (no itch/pain at all) to 10 (worst itch/pain ever experienced).

### EEG pre-processing

EEG data were pre-processed using FieldTrip (v.20191025;^54^) in MATLAB v.R2018b (The Mathworks Inc., Massachusetts, USA). Enabling comparison to relevant pain literature^15,43,55^, data were re-referenced to the common average. Data were band-pass filtered (1–80 Hz) and then notch-filtered (50 Hz) to remove line noise. While blinded to participant grouping, 2-s epochs containing electrode, muscle, or motion artefacts were visually rejected. Noisy channels (i.e. consistent low-quality signal or eight or more signal deviations^50^) were removed and interpolated using averaged triangular neighbours. The average number of channels interpolated was 1.55 (range 0–5) for resting state, 1.75 (range 0–5) for histamine stimulation, 1.29 (range 0–4) for electrical stimulation, and 1.07 (range 0–6) for CPT stimulation. An independent component analysis (ICA) was used to remove components representing blinks and saccades. Lastly, epochs still containing artefacts were visually rejected. Excluding participants with data missing from a condition, the average number of remaining epochs with EC was 85.57 at T1 (range: 58–90) and 84.86 at T2 (range: 21–90), and 86.63 at T1 (range: 62–90) and 86 at T2 (range: 58–90) with EO. The average number of remaining epochs was 67.68 (range: 59–75), 65.81 (range: 12–147), and 23.04 (range: 9–31) for the histamine, electrical, and CPT stimulation states, respectively. Minimum epoch numbers required in each analysis are described below.

### EEG analysis

Epochs were multiplied by a Hanning window and transformed using fast Fourier transform (1–45 Hz). For each participant, absolute power and peak frequencies were calculated across all electrodes (i.e. global) and for four regions of interest (ROIs)^56^ in a priori defined theta (4–7.5 Hz), alpha (8–12.5 Hz), and beta (13–30 Hz) frequency bands. ROIs were defined a priori as: frontal (Fp1, AF3, F7, F3, F4, F8, AF4, Fp2, Fz), central-temporal (FC1, FC5, T7, C3, C4, T8, FC6, FC2, Cz), parietal (CP1, CP5, P7, P3, Pz, P4, P8, CP6, CP2), and occipital (PO3, O1, Oz, O2, PO4). For stimulation states, the two hemispheres were differentiated to control for effects of contra- and ipsilateral stimulation. Compared to absolute means, centre of gravity (CoG) is a superior method of peak frequency calculation^42,57^, but only when narrow frequency bands can be defined^58^. As peak frequency ranges of chronic itch patients are currently undefined, peak frequencies were calculated using both methods (i.e. CoG and mean peak frequency) across the wide bands described above. In order to have sufficient data for the analyses, at least 60 epochs of signal for resting state or histamine (i.e. 2 min: sufficient for power and peak frequency analysis^59,60^), and 10 epochs for electrical and CPT stimulations (i.e. 20 s: sufficient for power only) were required. Sample sizes used for each analysis are detailed within the results.

### Statistical analysis

Frequentist analyses were conducted using IBM SPSS Statistics 25 (IBM Corp., Armonk, NY, USA). Intra-class correlations (ICC) indicated that, within individuals, resting state measures of CoG and mean peak frequency were strongly correlated, as were resting state measures of mean peak at T1 and T2 (Supplementary Information A). Thus, only analyses of T1 and mean peaks are reported.

For global and ROI resting state data, separate repeated-measures analyses of variance (RM-ANOVAs) assessed impact of group (i.e. patient/HC) and condition (i.e. EC/EO) on participants’ resting state power and peak frequencies (theta, alpha, beta). Theta-alpha ratios were calculated using the average power within each band during rest and compared between groups using an independent-samples t-test. Spearman’s rank correlation coefficients assessed relationships between EEG measures and patient characteristics (i.e. duration of chronic itch in years, TBSA, baseline pain and itch, and PTS symptoms reflected by total IES score). Post-hoc correlation coefficients were calculated between age and the EEG measures for the two groups separately as well as between age and disease duration.

For stimulation data, global averages of power and peak frequency were compared between groups using independent-samples t-tests. Additionally, 4 × 2 × 2 RM-ANOVAs assessed the impact of ROI (i.e. frontal/central-temporal/parietal/occipital) and hemisphere (i.e. ipsi-/contralateral to stimulation side) as within-participant factors, and group (i.e. patient/HC) as a between-participants factor, separately for power and peak frequencies in the theta, alpha, and beta bands. Pairwise comparisons of Bonferroni-adjusted estimated marginal means were used for post-hoc comparisons.

Normality was checked with skewness and kurtosis values, the Shapiro–Wilk normality test, and by visually inspecting the data in quantile–quantile-plots and boxplots. Variables with normality violations or extreme outliers were square root transformed. If outliers remained after transformation, analyses were conducted with and without outliers and their impact reported. A Mann–Whitney U test was used to analyse the alpha power during electrical stimulation, due to t-test assumption violations. Sphericity was investigated using Mauchly’s test for electrical, histamine, and CPT stimulation or Levene’s test for resting state; Greenhouse–Geisser corrected outcomes are reported when sphericity could not be assumed. Levene’s test assessed homogeneity for t-tests. For analyses including within- and between-participant factors, generalised η^2^ was calculated^61^; otherwise, partial η^2^ is reported.

Default settings in JASP (Version 0.14.1)^62^ were used to conduct Bayesian analyses on the global EEG outcomes during rest and stimulations. Bayes factors (BF) describe the likelihood of the alternative and null hypotheses. A BF_10_ between 3 and 20 is considered ‘positive’, 20–150 is ‘strong’, and > 150 is ‘very strong’ evidence for the alternative. For instance, a two-sided BF_10_ of 4 suggests that the data are 4 times more likely to occur under the alternative hypothesis than the null. A BF_10_ between 1/3 (i.e. 0.33) and 3 is considered ‘equivocal’, suggesting the evidence is inconclusive^63^. A BF_10_ between 1/20 and 1/3 (i.e. 0.05–0.33) is considered ‘positive’, 1/150–1/20 (i.e. 0.0067–0.05) is ‘strong’, and < 1/150 (i.e. < 0.0067) is ‘very strong’ evidence for the null hypothesis.

Unless stated, data are presented as arithmetic means ± standard deviations (SD), statistical tests were two-tailed, and significance was recognised at *p* < 0.05.

## Supplementary Information

Supplementary Information.

## Data Availability

Data and scripts for data preprocessing and analyses will be made available via a complete publication data package in the DataverseNL repository via https://dataverse.nl/ upon publication of the study according to Leiden University policy. Upon request, all supporting data are available to Editorial Board Members and referees at the time of submission.
