# Supplementary material for: No indications for altered EEG oscillatory activity in patients with chronic post-burn itch compared to healthy controls
Source: Sci Rep. 2022 Mar 25;12:5184. doi: 10.1038/s41598-022-08742-8 (PMC8956573; doi:10.1038/s41598-022-08742-8)
Supplement: Supplementary file 1 — Supplementary Information. [file 41598_2022_8742_MOESM1_ESM.pdf]

Supplementary information regarding:

No indications for altered EEG oscillatory activity in patients with chronic post-burn itch compared to healthy controls

Authors: Samantha K. Millard, Klara Bokelmann, Rik Schalbroeck, Nic J.A. van der Wee, Nancy E.E. van Loey, Antoinette I.M. van Laarhoven

Index:

Supplementary Information A: intra-class correlations
Supplementary Information B: Boxplots comparing the patients and healthy controls on the theta, alpha, and beta frequency bands (global activity)
Supplementary Information C: Resting state region of interest analyses
Supplementary Information D: Exploratory histamine analysis
Supplementary Information E: Exploratory electrical “must-scratch” threshold analysis
Supplementary Information F: Exploratory painful cold pressor task analysis

Supplementary Information A

Intra-class correlations

Intra-class correlations (ICC) confirmed that, over the entire sample of participants, resting state measures of centre of gravity (CoG) and mean peak were significantly, and generally strongly, correlated at the beginning (T1) of the experiment (Table S-A.1). In addition, the mean peak frequency outcomes were also strongly correlated at T1 and the end of the experiment (T2; Table S-A.2). Thus, only analyses of T1 and mean peaks are reported in the main article.

Table S-A.1 – Means and standard deviation (SD) of mean peak (MP) frequency and centre of gravity (CoG) peak frequency methodologies as well as their intraclass correlations (ICC) during eyes closed (EC) and eyes open (EO) resting state measures per group of patients with chronic post-burn itch and healthy controls (HCs) at the beginning (T1) and the end (T2) of the experiment. Sample sizes of the patients were *n* = 14 for EC1 and EO2; *n* = 13 for EO1; *n* = 15 for EC2. Sample sizes of the HCs were *n* = 14 for EC1 and EO1; *n* = 13 for EC2 and EO2. CI = confidence interval. df = degrees of freedom. * $p < .05$. ** $p < .01$. *** $p < .001$

Group	Frequency band	Eyes open/ closed	Mean CoG (SD)	Mean MP (SD)	ICC	95% - CI	*F* (df1, df2)	*p*-value
Patients	Theta	EC1	5.71 (.17)	5.67 (.35)	.88	.62; .96	7.90 (13,13)	<.001***
		EO1	5.63 (.15)	5.50 (.31)	.82	.28; .95	8.02 (12, 12)	.001**
		EC2	5.77 (.19)	5.78 (.39)	.89	.67; .96	8.64 (14,14)	<.001***
		EO2	5.66 (.18)	5.56 (.37)	.85	.52; .95	7.90 (13,13)	<.001***
	Alpha	EC1	10.07 (.38)	10.04 (.57)	.96	.87; .99	22.41 (13,13)	<.001***
		EO1	10.12 (.19)	10.01 (.32)	.89	.49; .97	13.12 (12,12)	<.001***
		EC2	10.05 (.43)	10.01 (.62)	.96	.90; .99	27.25 (14,14)	<.001***
		EO2	10.05 (.23)	9.89 (.38)	.87	.22; .97	13.67 (13,13)	<.001***
	Beta	EC1	19.27 (.55)	17.19 (1.00)	.28	-.08; .71	11.32 (13,13)	.002**
		EO1	19.81 (.59)	17.99 (.98)	.37	-.07; .79	9.37 (12,12)	<.001***
		EC2	19.47 (.74)	17.71 (1.17)	.46	-.12; .83	8.24 (14,14)	<.001***
		EO2	19.81 (.59)	18.04 (1.00)	.39	-.08; .80	9.51 (13,13)	.002**

HCs	Theta	EC1	5.78	5.79	.91	.72; 97	10.64	<.001***
			(.30)	(.56)			(13,13)	
		EO1	5.66	5.56	.87	.60; .96	9.09	<.001***
			(.21)	(.42)			(13,13)	
		EC2	5.82	5.86	.91	.71; .97	10.66	<.001***
			(.28)	(.53)			(12,12)	
		EO2	5.72	5.67	.91	.71; .97	10.70	<.001***
			(.26)	(.48)			(12,12)	
	Alpha	EC1	9.96	9.81	.94	.69; .98	22.51	<.001***
			(.39)	(.56)			(13,13)	
		EO1	10.10	9.96	.92	.67; .98	16.11	<.001***
			(.32)	(.52)			(13,13)	
		EC2	9.92	9.75	.93	.57; .98	25.69	<.001***
			(.38)	(.55)			(12,12)	
		EO2	10.03	9.86	.92	.53; .98	20.10	<.001***
			(.35)	(.55)			(12,12)	
	Beta	EC1	19.00	16.85	.29	-.05; .71	8.45	<.001***
			(.62)	(.92)			(13,13)	
		EO1	19.61	17.62	.45	-.07; .83	12.31	<.001***
			(.74)	(1.22)			(13,13)	
		EC2	18.98	16.97	.38	-.08; .79	9.09	<.001***
			(.72)	(1.08)			(12,12)	
		EO2	19.67	17.81	.50	-.07; .86	14.77	<.001***
			(.81)	(1.22)			(12,12)	

Table S-A.2 – Means and standard deviation (SD) of mean peak (MP) frequency measures at the beginning (T1) and end (T2) of the experiment as well as their intraclass correlations (ICC) separately for eyes closed (EC) and eyes open (EO) resting state and for the patients and healthy controls (HCs). Sample sizes of the patients were n = 14 for EC and n = 12 for EO. Sample sizes of the HCs were n = 13. CI = confidence interval. df = degrees of freedom. * $p < .05$. ** $p < .01$. *** $p < .001$

Group	Frequency band	Eyes open/ closed	Mean MP T1 (SD)	Mean MP T2 (SD)	ICC	95% - CI	*F* (df1, df2)	*p*-value
Patients	Theta	EC	5.67 (.35)	5.78 (.39)	.96	.68; .99	39.29 (13,13)	<.001***
		EO	5.50 (.31)	5.56 (.37)	.97	.91; .99	38.88 (11,11)	<.001***
	Alpha	EC	10.04 (.57)	10.01 (.62)	.97	.90; .99	29.82 (13,13)	<.001***
		EO	10.01 (.32)	9.89 (.38)	.94	.79; .98	15.25 (11,11)	<.001***
	Beta	EC	17.19 (1.00)	17.71 (1.17)	.74	.23; .91	3.98 (13,13)	.009**
		EO	17.99 (.98)	18.04 (1.00)	.93	.76; .98	13.79 (11,11)	<.001***
HCs	Theta	EC	5.79 (.56)	5.86 (.53)	.99	.96; 1.00	98.47(12,12)	<.001***
		EO	5.56 (.42)	5.67 (.48)	.98	.81; 1.00	93.43 (12,12)	<.001***
	Alpha	EC	9.81 (.56)	9.75 (.55)	.98	.94; .99	52.26 (12,12)	<.001***
		EO	9.96 (.52)	9.86 (.55)	.97	.91; .99	42.40 (12,12)	<.001***
	Beta	EC	16.85 (.92)	16.97 (1.08)	.88	.62; .96	8.30 (12,12)	<.001***
		EO	17.62 (1.22)	17.81 (1.22)	.90	.69; .97	9.88 (12,12)	<.001***

Supplementary Information B

Boxplots comparing the patients and healthy controls on the theta, alpha, and beta frequency bands (global activity)

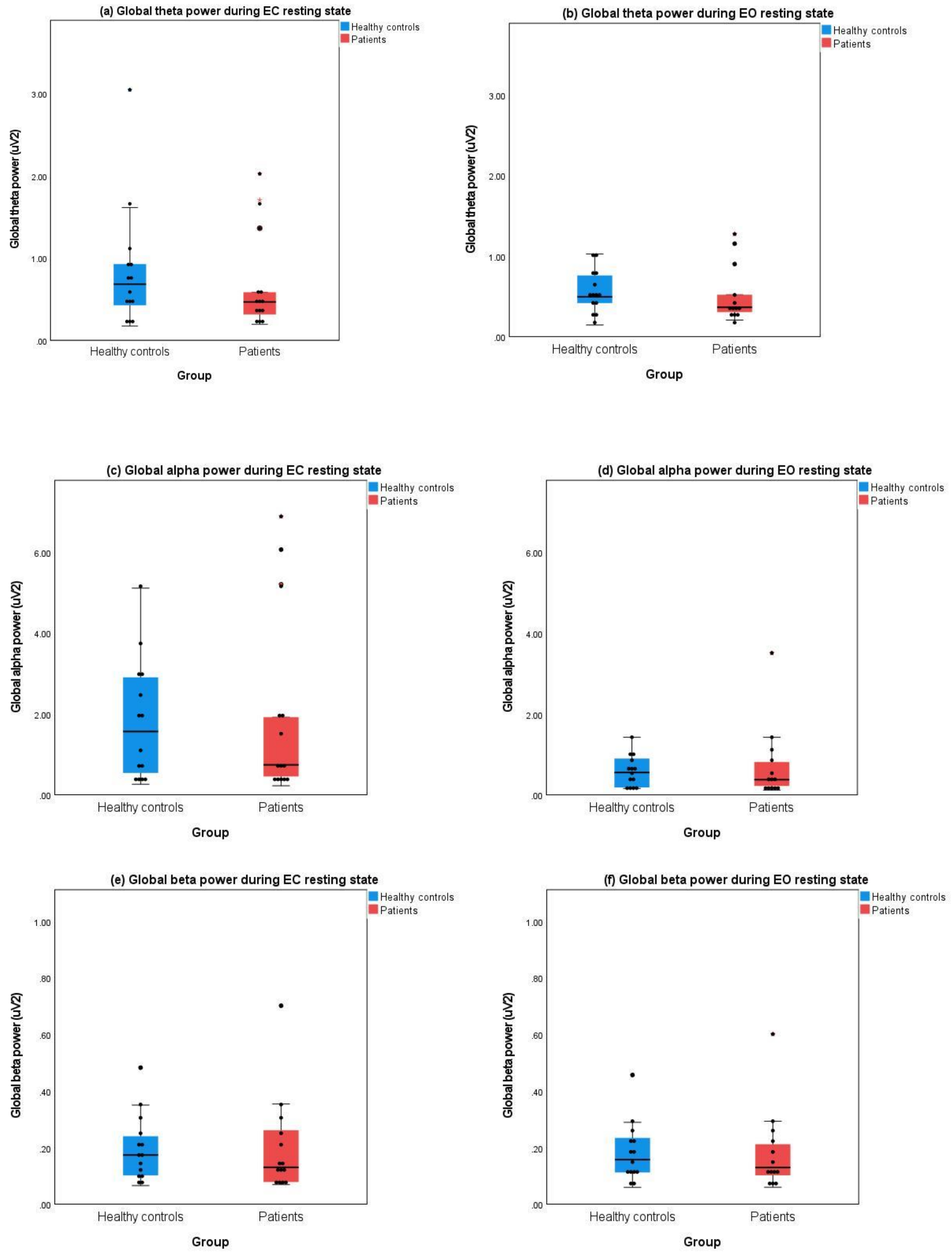

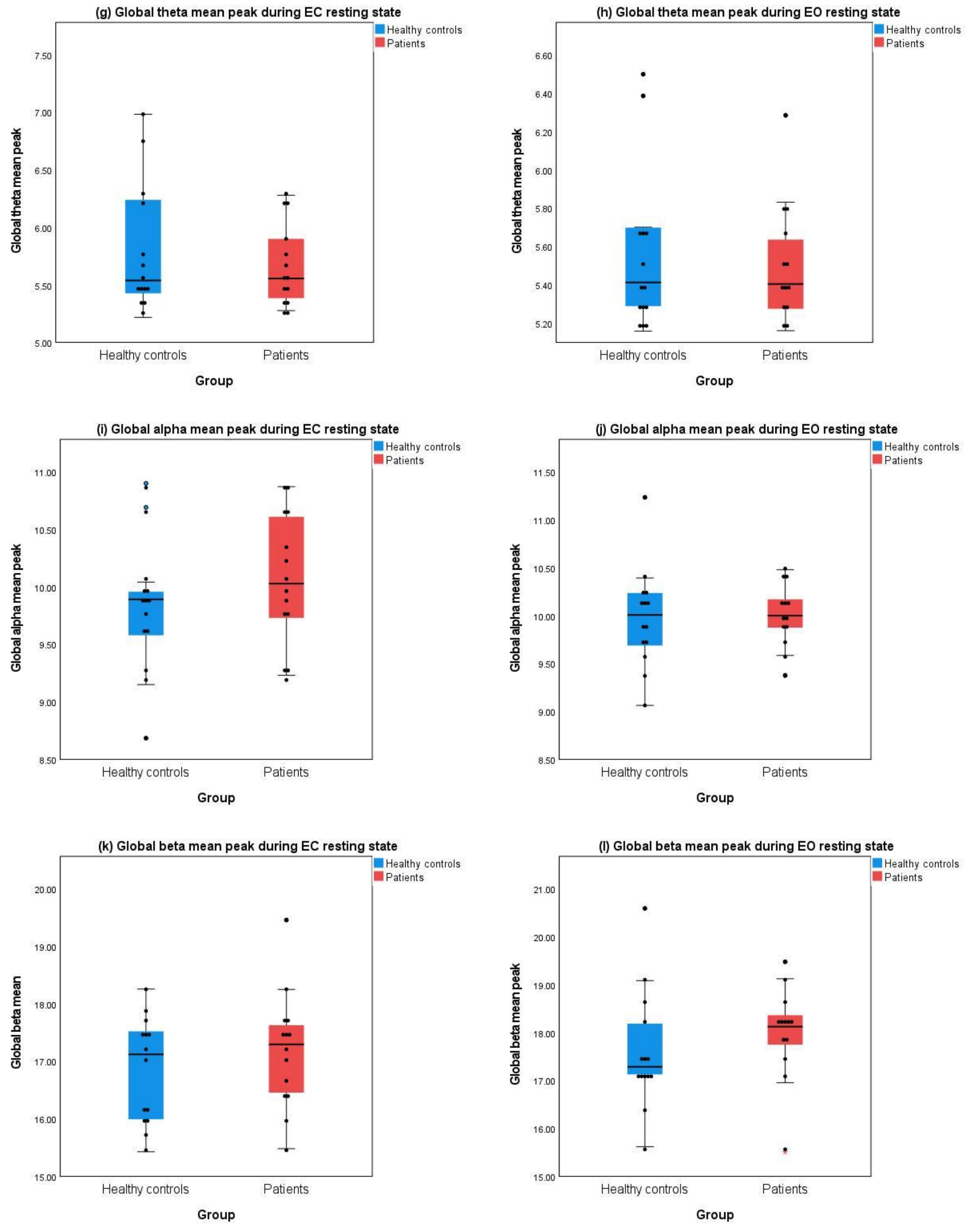

Figure S-B.1 – Boxplots depicting global power (panels a to f) and mean peak frequency (panels g to l) during eyes closed (EC) and eyes open (EO) resting states for patients with chronic post-burn itch (in red) and healthy controls (in blue).

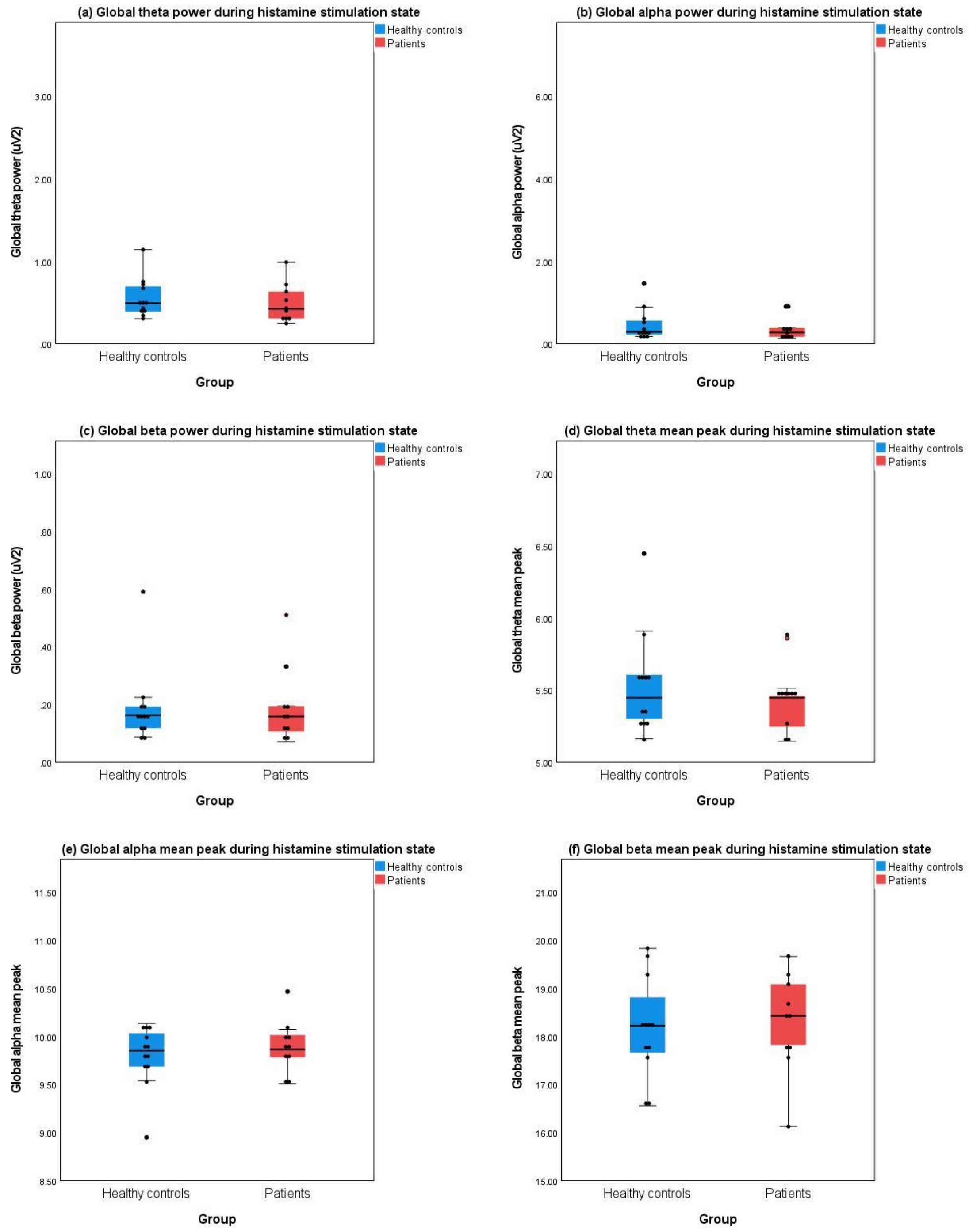

Figure S-B.2 – Boxplots depicting global power (panels a to c) and mean peak frequency (panels d to during histamine stimulation for patients with chronic post-burn itch (in red) and healthy controls (in blue).

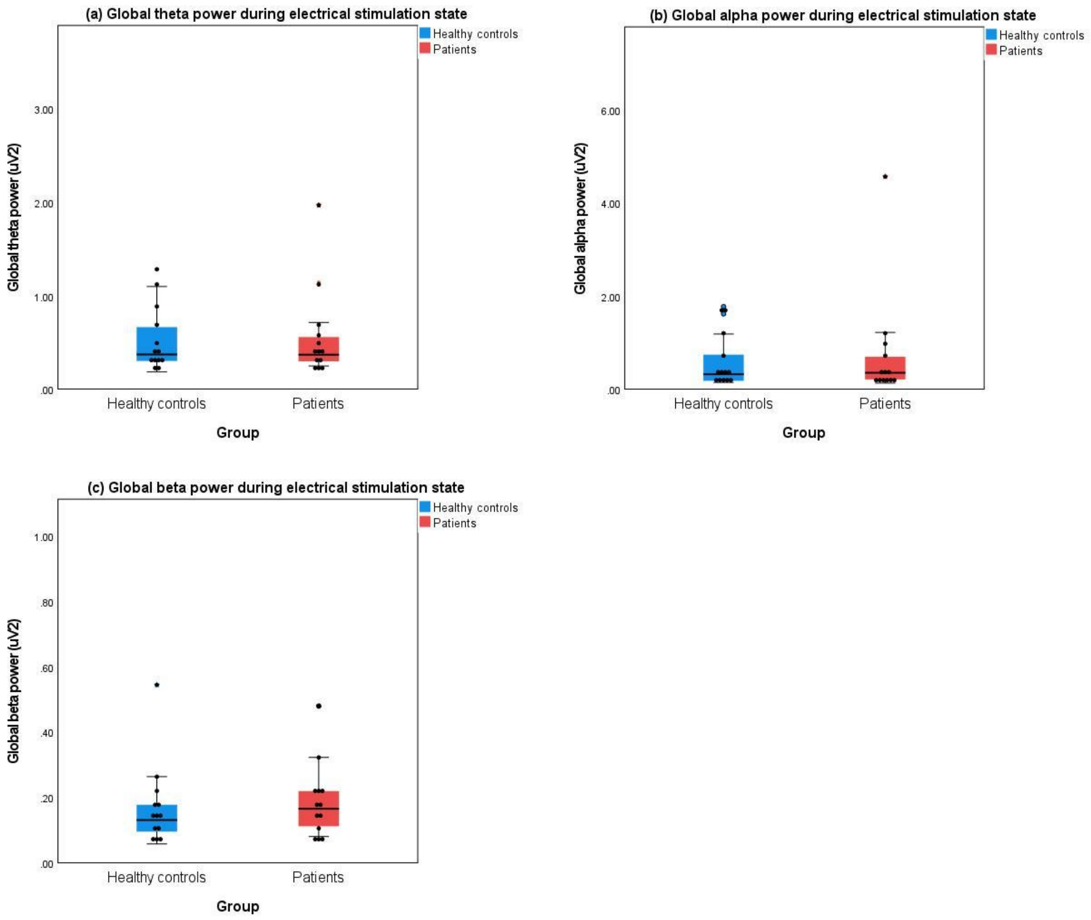

Figure S-B.3 – Boxplots depicting global power (panels a to c) during electrical stimulation for patients with chronic post-burn itch (in red) and healthy controls (in blue).

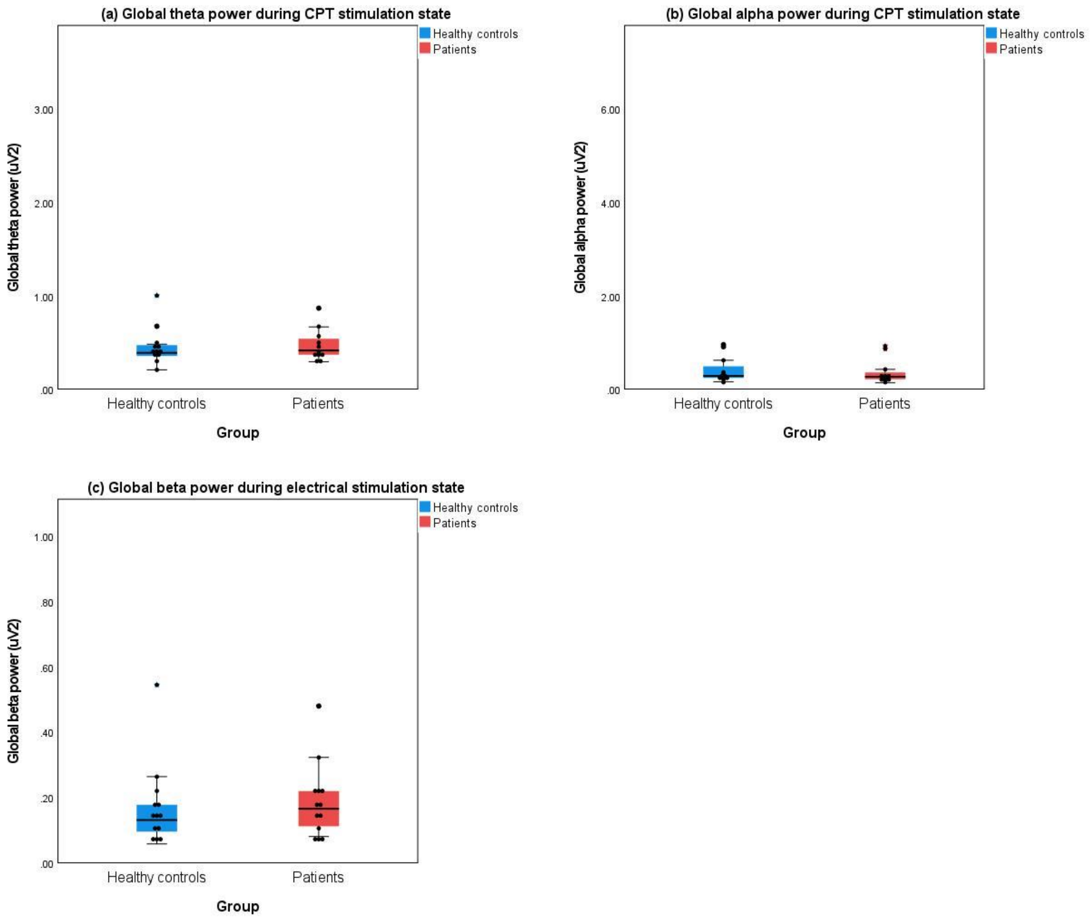

Figure S-B.4 – Boxplots depicting global power (panels a to c) during the cold pressor task (CPT) for patients with chronic post-burn itch (in red) and healthy controls (in blue).

Supplementary Information C

Resting state region of interest analyses

Four separate region of interest (ROI) repeated-measures analyses of variance (RM-ANOVAs) assessed the impact of group (patients with chronic post-burn itch/healthy controls) and condition (eyes open/eyes closed) on participants' resting state power and peak frequencies in theta, alpha, and beta frequency bands. ROIs were defined as: frontal (Fp1, AF3, F7, F3, F4, F8, AF4, Fp2, Fz), central-temporal (FC1, FC5, T7, C3, C4, T8, FC6, FC2, Cz), parietal (CP1, CP5, P7, P3, Pz, P4, P8, CP6, CP2), and occipital (PO3, O1, Oz, O2, PO4). For each ROI there were no main or interaction effects involving group. There were significant main effects of condition on power in all frequency bands (Table S-C.1), with higher power when eyes were closed than when open. With regards to mean peak frequency, there was a main effect of condition on the theta mean peak frequency for each ROI, with higher theta frequencies when eyes were closed than when open. There was also a main effect of condition on the beta mean peak frequency for the frontal, central-temporal, and parietal ROIs, with higher beta peak frequencies when eyes were open than when closed (Table S-C.2).

Table S-C.1 - Comparison of electroencephalography (EEG) mean (standard deviation; SD) power outcomes between patients with chronic post-burn itch and healthy controls (HCs) during eyes closed (EC) and eyes open (EO) resting states for separate regions of interest (ROIs). Sample sizes of the patients were n = 14 for frontal and central-temporal ROIs, n = 15 for parietal and occipital ROIs. Sample sizes of the HCs were n = 13 for frontal and central-temporal ROIs, n = 14 for parietal and occipital ROIs. For theta and alpha bands, the displayed means are based on the untransformed variables; the analysis of variance was conducted using square-root transformed variables. The ROI electrodes refer to: frontal = Fp1, AF3, F7, F3, F4, F8, AF4, Fp2, Fz; central-temporal = FC1, FC5, T7, C3, C4, T8, FC6, FC2, Cz; parietal = CP1, CP5, P7, P3, Pz, P4, P8, CP6, CP2; occipital = PO3, O1, Oz, O2, PO4. df = degrees of freedom. $\eta_G{}^2$ = generalised eta squared. * $p < .05$. ** $p < .01$. *** $p < .001$.

Frequency band		Mean power (SD) patients		Mean power (SD) HCs		Condition (EC/EO)			Group (patient/HC)			Condition * group		
	ROI	EC	EO	EC	EO	F (df1, df2)	p-value	ηG^2	F (df1, df2)	p-value	ηG^2	F (df1, df2)	p-value	ηG^2
Theta	Frontal	.72 (.45)	.60 (.34)	.74 (.86)	.48 (.31)	7.89 (1, 25)	.009**	.03	.38 (1, 25)	.545	.01	.44 (1, 25)	.515	<.01
	Central-temporal	.72 (.45)	.60 (.34)	.74 (.86)	.48 (.31)	7.89 (1, 25)	.009**	.03	.38 (1, 25)	.545	.01	.44 (1, 25)	.515	<.01
	Parietal	.76 (.59)	.53 (.35)	.71 (1.00)	.38 (.31)	10.27 (1, 27)	.003**	.05	.775 (1, 27)	.387	.02	.01 (1, 27)	.908	<.01
	Occipital	1.06 (.74)	.73 (.40)	.91 (1.07)	.57 (.43)	10.59 (1, 27)	.003**	.05	1.05 (1, 27)	.316	.03	.07 (1, 27)	.791	<.01

Alpha	Frontal	1.26	.54	1.81	.67	30.53	<.001***	.15	.24	.628	.01	.48	.496	<.01
		(1.31)	(.42)	(1.90)	(.85)	(1, 25)			(1, 25)			(1, 25)		
	Central-	1.26	.54	1.81	.67	30.53	<.001***	.15	.24	.628	.01	.48	.496	<.01
	temporal	(1.31)	(.42)	(1.90)	(.85)	(1, 25)			(1, 25)			(1, 25)		
	Parietal	1.94	.61	1.78	.67	32.93	<.001***	.16	.01	.918	<.001	.06	.805	<.01
		(2.33)	(.54)	(1.82)	(.91)	(1, 27)			(1, 27)			(1, 27)		
	Occipital	3.80	.83	3.85	.93	38.07	<.001***	.24	<.001	.983	<.001	.01	.943	<.01
		(4.66)	(.65)	(4.00)	(1.16)	(1, 27)			(1, 27)			(1, 27)		
Beta	Frontal	.19	.24	.15	.20	4.44	.045*	.03	.52	.479	.02	.03	.868	<.01
		(.16)	(.18)	(.09)	(.16)	(1, 25)			(1, 25)			(1, 25)		
	Central-	.18	.24	.15	.20	4.44	.045*	.03	.52	.479	.02	.03	.868	<.01
	temporal	(.16)	(.18)	(.09)	(.16)	(1, 25)			(1, 25)			(1, 25)		
	Parietal	.23	.18	.16	.11	15.24	.001**	.03	1.74	.198	.06	.04	.845	<.01
		(.21)	(.14)	(.09)	(.05)	(1, 27)			(1, 27)			(1, 27)		
	Occipital	.34	.23	.22	.14	18.57	<.001***	.07	2.28	.143	.07	.35	.561	<.01
		(.29)	(.18)	(.14)	(.06)	(1, 27)			(1, 27)			(1, 27)		

Table S-C.2 - Comparison of electroencephalography (EEG) mean peak frequency outcomes between patients with chronic post-burn itch (*n* = 14) and healthy controls (HCs; *n* = 13) during eyes closed (EC) and eyes open (EO) resting states for separate regions of interest (ROIs) For the frontal and central-temporal ROIs in the alpha band, the displayed means are based on the untransformed variables; the analysis of variance was conducted using square-root transformed variables. The ROI electrodes refer to: frontal = Fp1, AF3, F7, F3, F4, F8, AF4, Fp2, Fz; central-temporal = FC1, FC5, T7, C3, C4, T8, FC6, FC2, Cz; parietal = CP1, CP5, P7, P3, Pz, P4, P8, CP6, CP2; occipital = PO3, O1, Oz, O2, PO4. SD = standard deviation. $\eta_G{}^2$ = generalised eta squared.

Frequency band		Mean peak frequency (SD) patients		Mean peak frequency (SD) HCs		Condition (EC/EO)			Group (patient/HC)			Condition * group		
	ROI	EC	EO	EC	EO	F (1, 25)	*p*-value	ηG^2	F (1, 25)	*p*-value	ηG^2	F (1, 25)	*p*-value	ηG^2
Theta	Frontal	5.71 (.40)	5.55 (.35)	5.69 (.52)	5.54 (.40)	18.49	<.001***	.04	.02	.878	<.01	.10	.759	<.01
	Central-temporal	5.66 (.43)	5.49 (.30)	5.67 (.53)	5.46 (.45)	23.48	<.001***	.05	.03	.867	<.01	.01	.928	<.01
	Parietal	5.76 (.47)	5.56 (.36)	5.81 (.52)	5.56 (.41)	23.58	<.001***	.07	<.01	.949	<.01	.06	.802	<.01
	Occipital	5.84 (.49)	5.56 (.36)	5.78 (.46)	5.55 (.38)	22.02	<.001***	.10	.12	.736	<.01	.58	.453	<.01
Alpha	Frontal	9.82 (.65)	9.90 (.40)	10.02 (.48)	10.01 (.51)	.39	.539	<.01	.91	.348	.03	.61	.442	.01
	Central-temporal	9.86 (.61)	9.92 (.38)	9.89 (.42)	10.00 (.46)	2.20	.151	.02	.24	.629	.01	<.01	.979	<.01

	Parietal	9.89 (.68)	9.97 (.43)	9.98 (.47)	10.11 (.47)	2.52	.125	.02	.60	.444	.02	<.01	.974	<.01
	Occipital	10.00 (.84)	9.94 (.46)	10.09 (.49)	10.07 (.49)	<.01	.952	<.01	.65	.429	.02	.04	.849	<.01
Beta	Frontal	17.11 (.98)	18.67 (1.59)	17.53 (1.27)	18.97 (1.90)	24.60	<.001***	.23	.71	.408	.02	.10	.758	<.01
	Central-temporal	17.08 (.81)	17.97 (1.12)	17.35 (1.27)	18.15 (1.41)	17.63	<.001***	.12	.29	.597	.01	.03	.861	<.01
	Parietal	16.71 (.85)	17.00 (.78)	16.79 (1.16)	17.18 (1.09)	6.90	.015*	.04	.23	.635	.01	.02	.877	<.01
	Occipital	16.51 (1.13)	16.48 (.95)	16.84 (1.13)	17.09 (1.04)	.84	.369	.01	2.35	.138	.07	.15	.699	<.01

.

Supplementary Information D

Exploratory histamine itch analysis

Table S-D.1 summarises all mean values for power and mean peaks per ROI during histamine itch stimulation across frequency bands.

Power

For histamine stimulation when including outliers (Table S-D.2), no significant main or interaction effects of group (patients with chronic post-burn itch/healthy controls) or stimulation side (ipsi-/contralateral) on power were found in any frequency band. However, significant main effects of ROI were found in the theta, alpha, and beta bands (see Table S-D.3 for post-hoc pairwise comparisons).

In the sensitivity analyses, after excluding one outlier from the alpha band RM-ANOVA, the main effect of group (patients with chronic post-burn itch/healthy controls) on alpha power remained nonsignificant ($F(1, 19) = .42$, $p = .523$, $\eta G^2 = .02$). Also, when excluding two outliers for the beta band, the main effect of group was still nonsignificant ($F(1, 18) = .45$, $p = .513$, $\eta G^2 = .02$).

Mean Peak Frequency

RM-ANOVAS did not find significant interaction or main effects of group (patients with chronic post-burn itch/healthy controls), ROI, or stimulation side (ipsi-/contralateral) on mean peak in the theta or alpha bands (Table S-D.4). However, in the beta band there was a significant main effect of ROI on mean beta peak frequency (see TableS-D.5 for post-hoc pairwise comparisons).

Table S-D.1 - Mean power and mean peak (MP) values and standard deviations (SD) in patients with chronic post-burn itch (*n* = 10) and healthy controls (HCs; *n* = 12) during histamine stimulation for separate regions of interest (ROIs). These values were calculated from untransformed data while the outcomes of the analyses displayed in the following tables were based on square-root transformed data. The ROI electrodes refer to: frontal: left = Fp1, AF3, F7, F3, Fz; right = F4, F8, AF4, Fp2, Fz; central-temporal: left = FC1, FC5, T7, C3, Cz; right = C4, T8, FC6, FC2, Cz; parietal: left = CP1, CP5, P7, P3, Pz; right = P4, P8, CP6, CP2, Pz; occipital: left = PO3, O1, Oz; right = O2, PO4, Oz. Whether the left or right hemisphere were ipsi- or contralateral to the stimulation side differed across participants due to randomisation of stimulation side.

Frequency band	ROI	Stimulation side	Mean power (SD) patients	Mean power (SD) HCs	Mean MP (SD) patients	Mean MP (SD) HCs
Theta	Frontal	Contralateral	.53 (.28)	.64 (.32)	5.42 (.26)	5.54 (.38)
		Ipsilateral	.45 (.21)	.60 (.25)	5.43 (.19)	5.51 (.36)
	Central-temporal	Contralateral	.41 (.22)	.48 (.24)	5.34 (.23)	5.49 (.30)
		Ipsilateral	.43 (.18)	.44 (.22)	5.34 (.20)	5.51 (.35)
	Parietal	Contralateral	.59 (.36)	.66 (.36)	5.47 (.32)	5.55 (.39)
		Ipsilateral	.45 (.33)	.49 (.29)	5.42 (.28)	5.59 (.45)
	Occipital	Contralateral	.85 (.39)	.84 (.45)	5.51 (.16)	5.49 (.34)
		Ipsilateral	.71 (.52)	.81 (.54)	5.49 (.12)	5.57 (.37)
Alpha	Frontal	Contralateral	.34 (.27)	.44 (.31)	9.79 (.16)	9.73 (.36)
		Ipsilateral	.30 (.20)	.42 (.30)	9.80 (.24)	9.72 (.37)
	Central-temporal	Contralateral	.30 (.25)	.37 (.31)	9.85 (.36)	9.79 (.31)
		Ipsilateral	.30 (.21)	.36 (.37)	9.85 (.34)	9.76 (.30)
	Parietal	Contralateral	.51 (.46)	.56 (.43)	9.94 (.34)	9.85 (.36)
		Ipsilateral	.39 (.36)	.49 (.53)	9.97 (.36)	9.82 (.33)
	Occipital	Contralateral	.63 (.46)	.60 (.34)	9.77 (.28)	9.78 (.44)
		Ipsilateral	.59 (.53)	.66 (.45)	9.88 (.30)	9.81 (.41)
Beta	Frontal	Contralateral	.24 (.22)	.22 (.14)	19.28 (1.39)	18.74 (1.57)
		Ipsilateral	.19 (.12)	.23 (.23)	19.29 (1.62)	18.98 (1.58)
	Central-temporal	Contralateral	.15 (.13)	.15 (.09)	18.44 (1.05)	18.12 (1.10)
		Ipsilateral	.16 (.10)	.15 (.10)	18.36 (1.08)	18.61 (1.07)
	Parietal	Contralateral	.20 (.16)	.20 (.18)	17.40 (.97)	17.47 (.97)
		Ipsilateral	.16 (.15)	.15 (.11)	17.51 (1.06)	17.58 (.95)
	Occipital	Contralateral	.27 (.25)	.23 (.17)	17.41 (1.34)	17.42 (1.35)
		Ipsilateral	.25 (.20)	.25 (.22)	17.49 (1.21)	17.32 (1.12)

Table S-D.2 Comparison of electroencephalography (EEG) power outcomes between patients with chronic post-burn itch (*n* = 10) and healthy controls (HCs; *n* = 12) during histamine stimulation for separate regions of interest (ROIs). All variables were square-root transformed before performing these analyses. The ROI electrodes refer to: frontal: left = Fp1, AF3, F7, F3, Fz; right = F4, F8, AF4, Fp2, Fz; central-temporal: left = FC1, FC5, T7, C3, Cz; right = C4, T8, FC6, FC2, Cz; parietal: left = CP1, CP5, P7, P3, Pz; right = P4, P8, CP6, CP2, Pz; occipital: left = PO3, O1, Oz; right = O2, PO4, Oz. Whether the left or right hemisphere were ipsi- or contralateral to the stimulation side differed across participants due to randomisation of stimulation side. df = degrees of freedom. η_G^2 = generalised eta squared. * p < .05. ** p < .01. *** p < .001.

Frequency band	Stimulation side (ipsi-/contralateral)			ROI			Group			Stimulation side * group			ROI * group			Stimulation side * ROI			Stimulation side * ROI * group		
	F-value (*df1*, *df2*)	*p*-value	ηG^2	*F*-value (*df1*, *df2*)	*p*-value	ηG^2	*F*-value (*df1*, *df2*)	*p*-value	ηG^2	*F*-value (*df1*, *df2*)	*p*-value	ηG^2	*F*-value (*df1*, *df2*)	*p*-value	ηG^2	*F*-value (*df1*, *df2*)	*p*-value	ηG^2	*F*-value (*df1*, *df2*)	*p*-value	ηG^2
Theta	3.70 (1, 20)	.069	.02	30.50 (1.99, 39.72)	<.001***	.15	.40 (1, 20)	.532	.01	.04 (1, 20)	.841	<.01	.73 (1.99, 39.72)	.487	.01	2.91 (1.64, 32.80)	.078	.01	.78 (1.64, 32.80)	.445	<.01
Alpha	1.88 (1, 20)	.186	<.01	32.24 (2.09, 41.87)	<.001***	.10	.32 (1, 20)	.580	.01	.33 (1, 20)	.572	<.01	.59 (2.09, 41.87)	.567	<.01	2.97 (1.97, 39.45)	.063	<.01	1.04 (1.97, 39.45)	.363	<.01
Beta	2.15 (1, 20)	.159	.01	9.39 (1.83, 36.63)	.001 **	.06	<.01 (1, 20)	.975	<.01	.15 (1, 20)	.703	<.01	.18 (1.83, 36.63)	.820	<.01	1.56 (2.09, 41.86)	.221	<.01	.54 (2.09, 41.86)	.598	<.01

Table S-D.3 Post-hoc pairwise comparisons of electroencephalography (EEG) power outcomes at each region of interest (ROI) during histamine itch stimulation. All variables were square-root transformed before performing these analyses. The means and standard errors (SE) are post-hoc estimations based on the variables included in the repeated-measures analysis of variance. The ROI electrodes refer to frontal = Fp1, AF3, F7, F3, F4, F8, AF4, Fp2, Fz; central-temporal = FC1, FC5, T7, C3, C4, T8, FC6, FC2, Cz; parietal = CP1, CP5, P7, P3, Pz, P4, P8, CP6, CP2; occipital = PO3, O1, Oz, O2, PO4. * p <.05. ** p <.01. *** p < .001.

Frequency band	ROI (*mean*, *SE*)	Frontal	Central-temporal	Parietal
		Mean difference (*SE*), *p*-value		
Theta	Frontal (.73, .04)	-		
	Central-temporal (.65, .03)	|.08| (.02), .017**	-	
	Parietal (.71, .04)	|.02| (.02), 1.00	|.06| (.02), .034*	-
	Occipital (.86, .05)	|.14| (.03), .001**	|.22| (.03), <.001***	|.16| (.02), <.001***
Alpha	Frontal (.58, .04)	-		
	Central-temporal (.54, .04)	|.04| (.02), .278	-	
	Parietal (.65, .06)	|.07| (.02), .034*	|.11| (.02), <.001***	-
	Occipital (.75, .05)	|.17| (.03), <.001***	|.21| (.03), <.001***	|.10| (.02), <.001***
Beta	Frontal (.44, .03)	-		
	Central-temporal (.37, .02)	|.07| (.02), .013*	-	
	Parietal (.40, .03)	|.04| (.02), .484	|.03| (.02), .596	-
	Occipital (.47, .04)	|.03| (.03), 1.00	|.10| (.02), .001**	|.07| (.01), <.001***

Table S-D.4 Comparison of electroencephalography (EEG) mean peak frequency outcomes between patients with chronic post-burn itch (*n* = 10) and healthy controls (HCs; *n* = 12) during histamine stimulation for separate regions of interest (ROIs). All variables were square-root transformed before performing these analyses. The ROI electrodes refer to: frontal: left = Fp1, AF3, F7, F3, Fz; right = F4, F8, AF4, Fp2, Fz; central-temporal: left = FC1, FC5, T7, C3, Cz; right = C4, T8, FC6, FC2, Cz; parietal: left = CP1, CP5, P7, P3, Pz; right = P4, P8, CP6, CP2, Pz; occipital: left = PO3, O1, Oz; right = O2, PO4, Oz. Whether the left or right hemisphere were ipsi- or contralateral to the stimulation side differed across participants due to randomisation of stimulation side. df = degrees of freedom. η_G^2 = generalised eta squared. * $p < .05$. ** $p < .01$. *** $p < .001$.

Frequency band	Stimulation side (ipsi-/contralateral)			ROI			Group			Stimulation side * group			ROI * group			Stimulation side * ROI			Stimulation side * ROI * group		
	F-value (*df1*, *df2*)	*p*-value	ηG^2	*F*-value (*df1*, *df2*)	*p*-value	ηG^2	*F*-value (*df1*, *df2*)	*p*-value	ηG^2	*F*-value (*df1*, *df2*)	*p*-value	ηG^2	*F*-value (*df1*, *df2*)	*p*-value	ηG^2	*F*-value (*df1*, *df2*)	*p*-value	ηG^2	*F*-value (*df1*, *df2*)	*p*-value	ηG^2
Theta	.32 (1, 20)	.578	<.01	2.49 (2.02, 40.39)	.095	.01	.69 (1, 20)	.415	.03	1.65 (1, 20)	.213	<.01	1.16 (2.02, 40.39)	.323	.01	.47 (2.19, 43.72)	.647	<.01	1.60 (2.19, 43.72)	.212	<.01
Alpha	1.34 (1, 20)	.260	<.01	2.35 (2.20, 44.02)	.102	.02	.36 (1, 20)	.557	.01	4.25 (1, 20)	.052	<.01	.28 (2.20, 44.02)	.780	<.01	2.16 (3, 60)	.102	<.01	.16 (3, 30)	.924	<.01
Beta	1.05 (1, 20)	.318	<.01	27.85 (1.97, 39.40)	<.001***	.25	.07 (1, 20)	.792	<.01	.61 (1, 20)	.444	<.01	.50 (1.97, 39.40)	.609	.01	.56 (2.24, 44.87)	.594	<.01	1.99 (2.24, 44.87)	.144	<.01

Table S-D.5 - Post-hoc pairwise comparisons of regions of interest (ROIs) mean peak beta frequency during histamine itch stimulation. All variables were square-root transformed before performing these analyses. The means and standard errors (SE) are post-hoc estimations based on the variables included in the repeated-measures analysis of variance. The ROI electrodes refer to: frontal = Fp1, AF3, F7, F3, F4, F8, AF4, Fp2, Fz; central-temporal = FC1, FC5, T7, C3, C4, T8, FC6, FC2, Cz; parietal = CP1, CP5, P7, P3, Pz, P4, P8, CP6, CP2; occipital = PO3, O1, Oz, O2, PO4. SE = standard error. * $p < .05$. ** $p < .01$. *** $p < .001$.

ROI (*mean*, *SE*)	Frontal	Central-temporal	Parietal
	Mean difference (*SE*), *p*-value		
Frontal (4.36, .04)	-		
Central-temporal (4.29, .03)	|.08| (.02), .016*	-	
Parietal (4.18, .03)	|.18| (.03), <.001***	|.11| (.01), <.001***	-
Occipital (4.17, .03)	|.19| (.03), <.001***	|.12| (.02), <.001***	|.01| (.02), 1.00

Supplementary Information E

Exploratory electrical "must-scratch" threshold analysis

Table S-E.1 summarises untransformed mean values per ROI for power during electrical itch stimulation across frequency bands.

Theta Power

During electrical itch stimulation at participants' individual "must-scratch" threshold, an RM-ANOVA (Table S-E.2) suggested a significant main effect of ROI and stimulation side (ipsi-/ contralateral) on theta power, as well as an interaction effect between stimulation side and ROI for theta power (see Table S-E.3 for post-hoc pairwise comparisons). No main or interaction effects of group (patients with chronic post-burn itch/healthy controls) could be detected.

Alpha Power

An RM-ANOVA (Table S-E.2) suggested nonsignificant main and interaction effects of group (patients with chronic post-burn itch/healthy controls) but a significant main effect of ROI on alpha power (see Table S-E.4 for post-hoc pairwise comparisons).

When three outliers were removed for a sensitivity analysis, there was still no main effect of group on alpha power ($F(1, 21) < .01$, $p = .926$, $\eta G^2 < .01$).

Beta Power

An RM-ANOVA (Table S-E.2) suggested a significant main effect of ROI (see Table S-E.4 for post-hoc pairwise comparisons) but no significant main or interaction effects of group (patients with chronic post-burn itch/healthy controls) on beta power.

Table S-E.1 - Electroencephalography (EEG) mean power values and standard deviation (SD) in patients with chronic post-burn itch (*n* = 13) and healthy controls (HCs; *n* = 13) during electrical stimulation for separate regions of interest (ROIs). These values were calculated from untransformed data while the outcomes of the analyses displayed in the following tables were based on square-root transformed data. The ROI electrodes refer to: frontal: left = Fp1, AF3, F7, F3, Fz; right = F4, F8, AF4, Fp2, Fz; central-temporal: left = FC1, FC5, T7, C3, Cz; right = C4, T8, FC6, FC2, Cz; parietal: left = CP1, CP5, P7, P3, Pz; right = P4, P8, CP6, CP2, Pz; occipital: left = PO3, O1, Oz; right = O2, PO4, Oz. Whether the left or right hemisphere were ipsi- or contralateral to the stimulation side differed across participants.

Frequency band	ROI	Stimulation side	Mean power (SD) patients	Mean power (SD) HCs
Theta	Frontal	Contralateral	.61 (.60)	.61 (.41)
		Ipsilateral	.61 (.67)	.57 (.43)
	Central-temporal	Contralateral	.48 (.34)	.49 (.28)
		Ipsilateral	.46 (.37)	.43 (.32)
	Parietal	Contralateral	.65 (.47)	.64 (.41)
		Ipsilateral	.54 (.55)	.47 (.37)
	Occipital	Contralateral	.84 (.62)	.77 (.49)
		Ipsilateral	.75 (.71)	.64 (.42)
Alpha	Frontal	Contralateral	.72 (1.26)	.57 (.55)
		Ipsilateral	.71 (1.27)	.56 (.57)
	Central-temporal	Contralateral	.56 (.85)	.43 (.36)
		Ipsilateral	.61 (1.00)	.42 (.47)
	Parietal	Contralateral	.87 (1.08)	.76 (.70)
		Ipsilateral	.83 (1.49)	.66 (.77)
	Occipital	Contralateral	1.13 (1.70)	.70 (.48)
		Ipsilateral	.99 (1.25)	.88 (.86)
Beta	Frontal	Contralateral	.23 (.14)	.19 (.12)
		Ipsilateral	.23 (.15)	.19 (.13)
	Central-temporal	Contralateral	.14 (.09)	.14 (.09)
		Ipsilateral	.16 (.11)	.14 (.11)
	Parietal	Contralateral	.20 (.14)	.18 (.18)
		Ipsilateral	.15 (.12)	.15 (.14)
	Occipital	Contralateral	.25 (.16)	.20 (.16)
		Ipsilateral	.22 (.16)	.21 (.24)

Table S-E.2 - Comparison of electroencephalography (EEG) power outcomes between patients with chronic post-burn itch (*n* = 13) and healthy controls (HCs; *n* = 13) during electrical itch stimulation at participants' "must-scratch" threshold for separate regions of interest (ROIs). All variables were square-root transformed before performing these analyses. The ROI electrodes refer to: frontal: left = Fp1, AF3, F7, F3, Fz; right = F4, F8, AF4, Fp2, Fz; central-temporal: left = FC1, FC5, T7, C3, Cz; right = C4, T8, FC6, FC2, Cz; parietal: left = CP1, CP5, P7, P3, Pz; right = P4, P8, CP6, CP2, Pz; occipital: left = PO3, O1, Oz; right = O2, PO4, Oz. Whether the left or right hemisphere were ipsi- or contralateral to the stimulation side differed across participants due to randomisation of stimulation side. df = degrees of freedom. η_G^2 = generalised eta squared. * $p < .05$. ** $p < .01$. *** $p < .001$.

Frequency band	Stimulation side (ipsi-/contralateral)			ROI			Group			Stimulation side * group			ROI * group			Stimulation side * ROI			Stimulation side * ROI * group		
	F-value (*df1*, *df2*)	*p*-value	η_G^2	*F*-value (*df1*, *df2*)	*p*-value	η_G^2	*F*-value (*df1*, *df2*)	*p*-value	η_G^2	*F*-value (*df1*, *df2*)	*p*-value	η_G^2	*F*-value (*df1*, *df2*)	*p*-value	η_G^2	*F*-value (*df1*, *df2*)	*p*-value	η_G^2	*F*-value (*df1*, *df2*)	*p*-value	η_G^2
Theta	9.59 (1, 20)	.005**	.01	21.91 (3, 72)	<.001***	.05	.01 (1, 24)	.907	<.01	.38 (1, 24)	.543	<.01	.32 (3, 72)	.812	<.01	3.16 (2.11, 50.64)	.049*	<.01	.12 (2.11, 50.64)	.900	<.01
Alpha	3.16 (1, 24)	.088	<.01	31.78 (3, 72)	<.001***	.04	.06 (1, 24)	.802	<.01	.36 (1, 24)	.557	<.01	.34 (3, 72)	.793	<.01	2.03 (3, 72)	.117	<.01	1.43 (3, 72)	.242	<.01
Beta	3.46 (1, 24)	.075	<.01	8.47 (1.50, 35.93)	.002 **[2]	.05	.32 (1, 24)	.578	.01	.06 (1, 24)	.806	<.01	.36 (1.50, 58.64)	.640	<.01	2.11 (2.44, 58.64)	.120	<.01	.77 (2.44, 58.64)	.492	<.01

Table S-E.3 - Post-hoc pairwise comparisons of interaction effects between stimulation side and regions of interest (ROIs) on electroencephalography (EEG) theta power during electrical itch stimulation at participants' "must-scratch" threshold. All variables were square-root transformed before performing these analyses. The means and standard errors (SE) are post-hoc estimations based on the variables included in the repeated-measures analysis of variance. Whether the left or right hemisphere were ipsi- or contralateral to the stimulation side differed across participants due to randomisation of stimulation side. The ROI electrodes refer to: frontal = Fp1, AF3, F7, F3, F4, F8, AF4, Fp2, Fz; central-temporal = FC1, FC5, T7, C3, C4, T8, FC6, FC2, Cz; parietal = CP1, CP5, P7, P3, Pz, P4, P8, CP6, CP2; occipital = PO3, O1, Oz, O2, PO4. * $p < .05$. ** $p < .01$. *** $p < .001$.

ROI	Mean power (SE) Contralateral	Mean power (SE) Ipsilateral	Mean difference (contra - ipsilateral)	SE	*p*-value
Frontal	.74 (.05)	.71 (.06)	|.02|	.02	.215
Central-temporal	.67 (.04)	.63 (.04)	|.03|	.02	.137
Parietal	.76 (.05)	.66 (.05)	|.11|	.03	.004**
Occipital	.85 (.06)	.78 (.06)	|.07|	.03	.017*

Table S-E.4 - Post-hoc pairwise comparisons of region of interest (ROI) effects on electroencephalography (EEG) alpha and beta power during electrical itch stimulation at participants' "must-scratch" threshold. All variables were square-root transformed before performing these analyses. The means and standard errors (SE) are post-hoc estimations based on the variables included in the repeated-measures analysis of variance. The ROI electrodes refer to: frontal = Fp1, AF3, F7, F3, F4, F8, AF4, Fp2, Fz; central-temporal = FC1, FC5, T7, C3, C4, T8, FC6, FC2, Cz; parietal = CP1, CP5, P7, P3, Pz, P4, P8, CP6, CP2; occipital = PO3, O1, Oz, O2, PO4. * $p < .05$. ** $p < .01$. *** $p < .001$.

Frequency band	ROI (*mean*, *SE*)	Frontal	Central-temporal	Parietal
		Mean difference (*SE*), *p*-value		
Alpha	Frontal (.69, .08)	-		
	Central-temporal (.62, .07)	|.07| (.03), .112	-	
	Parietal (.77, .09)	|.08| (.02), .008**	|.14| (.03), <.001***	-
	Occipital (.85, .09)	|.16| (.02), <.001***	|.23| (.03), <.001***	|.09| (.02), <.001***
Beta	Frontal (.44, .03)	-		
	Central-temporal (.37, .02)	|.07| (.02), .003**	-	
	Parietal (.39, .03)	|.05| (.02), .289	|.02| (.01), .341	-
	Occipital (.44, .03)	|.01| (.03), 1.00	|.08| (.02), .001**	|.05| (.01), <.001***

Supplementary Information F

Exploratory painful cold pressor task analysis

Table S-F.1 summarises all mean values per ROI for power during cold pressor task (CPT) pain stimulation across frequency bands.

Theta power

During CPT pain stimulation, an RM-ANOVA (Table S-F.2) did not suggest main or interaction effects of group (patients with chronic post-burn itch/healthy controls) on theta power. However, the analysis did suggest a significant main effect of ROI on theta power and an interaction effect between ROI and stimulation side (ipsi-/contralateral) on theta power (see Table S-F.3 for post-hoc pairwise comparisons).

Excluding two outliers in the theta band, there the main effect of group on theta power was still nonsignificant ($F(1, 20) < .01$, $p = .950$, $\eta G^2 < .01$).

Alpha power

During CPT pain stimulation, an RM-ANOVA (Table S-F.2) did not suggest significant main or interaction effects of group (patients with chronic post-burn itch/healthy controls) on alpha power. However, the analysis suggested significant main effects of ROI and stimulation side (ipsi-/contralateral), as well as an interaction effect between ROI and stimulation side on alpha power (see Table S-F.3 for post-hoc pairwise comparisons).

Excluding two outliers in the alpha band, the RM-ANOVA for alpha power still showed nonsignificant effects of group on alpha power ($F(1, 20) < .01$, $p = .949$, $\eta G^2 < .01$).

Beta power

During CPT pain stimulation, an RM-ANOVA (Table S-F.2) did not suggest a significant main or interaction effects of group (patients with chronic post-burn itch /healthy controls) on beta power. However, the analysis suggested a significant main effect of ROI on beta power (see table S-F.4 for post-hoc pairwise comparisons).

Excluding one outlier from the analysis of beta power, the effect of group remained nonsignificant ($F(1, 21) < .01$ $p = .997$, $\eta G^2 < .01$).

Table S-F.1 – Electroencephalography (EEG) mean power values and standard deviation (SD) in patients with chronic post-burn itch (*n* = 12) and healthy controls (HCs; *n* = 12) during cold pressor task (CPT) stimulation for separate regions of interest (ROIs). These values were calculated from untransformed data while the outcomes of the analyses displayed in the following tables were based on square-root transformed data. The ROI electrodes refer to: frontal: left = Fp1, AF3, F7, F3, Fz; right = F4, F8, AF4, Fp2, Fz; central-temporal: left = FC1, FC5, T7, C3, Cz; right = C4, T8, FC6, FC2, Cz; parietal: left = CP1, CP5, P7, P3, Pz; right = P4, P8, CP6, CP2, Pz; occipital: left = PO3, O1, Oz; right = O2, PO4, Oz. Whether the left or right hemisphere were ipsi- or contralateral to the stimulation side differed across participants.

Frequency band	ROI	Stimulation side	Mean power (SD) patients	Mean power (SD) HCs
Theta	Frontal	Contralateral	.50 (.20)	.47 (.28)
		Ipsilateral	.52 (.20)	.51 (.25)
	Central-temporal	Contralateral	.37 (.15)	.42 (.24)
		Ipsilateral	.42 (.18)	.37 (.21)
	Parietal	Contralateral	.58 (.29)	.53 (.26)
		Ipsilateral	.41 (.25)	.39 (.23)
	Occipital	Contralateral	.74 (.32)	.64 (.29)
		Ipsilateral	.67 (.34)	.65 (.32)
Alpha	Frontal	Contralateral	.33 (.24)	.36 (.22)
		Ipsilateral	.32 (.22)	.37 (.19)
	Central-temporal	Contralateral	.30 (.21)	.40 (.32)
		Ipsilateral	.32 (.27)	.26 (.17)
	Parietal	Contralateral	.49 (.44)	.59 (.57)
		Ipsilateral	.36 (.34)	.35 (.25)
	Occipital	Contralateral	.55 (.40)	.72 (.73)
		Ipsilateral	.55 (.48)	.62 (.51)
Beta	Frontal	Contralateral	.26 (.28)	.20 (.16)
		Ipsilateral	.20 (.10)	.24 (.23)
	Central-temporal	Contralateral	.17 (.14)	.16 (.15)
		Ipsilateral	.18 (.10)	.15 (.11)
	Parietal	Contralateral	.22 (.20)	.18 (.13)
		Ipsilateral	.19 (.17)	.14 (.09)
	Occipital	Contralateral	.30 (.32)	.21 (.16)
		Ipsilateral	.26 (.24)	.21 (.13)

Table S-F.2 - Comparison of electroencephalography (EEG) power outcomes between patients with chronic post-burn itch (*n* = 12) and healthy controls (HCs; *n* = 12) during cold pressor task (CPT) stimulation for separate regions of interest (ROIs). All variables were square-root transformed before performing these analyses. The ROI electrodes refer to: frontal: left = Fp1, AF3, F7, F3, Fz; right = F4, F8, AF4, Fp2, Fz; central-temporal: left = FC1, FC5, T7, C3, Cz; right = C4, T8, FC6, FC2, Cz; parietal: left = CP1, CP5, P7, P3, Pz; right = P4, P8, CP6, CP2, Pz; occipital: left = PO3, O1, Oz; right = O2, PO4, Oz. Whether the left or right hemisphere were ipsi- or contralateral to the stimulation side differed across participants due to randomisation of stimulation side. df = degrees of freedom. η_G^2 = generalised eta squared. * $p < .05$. ** $p < .01$. *** $p < .001$.

Frequency band	Stimulation side (ipsi-/contralateral)			ROI			Group			Stimulation side * group			ROI * group			Stimulation side * ROI			Stimulation side * ROI * group		
	F-value (*df1*, *df2*)	*p*-value	ηG^2	*F*-value (*df1*, *df2*)	*p*-value	ηG^2	*F*-value (*df1*, *df2*)	*p*-value	ηG^2	*F*-value (*df1*, *df2*)	*p*-value	ηG^2	*F*-value (*df1*, *df2*)	*p*-value	ηG^2	*F*-value (*df1*, *df2*)	*p*-value	ηG^2	*F*-value (*df1*, *df2*)	*p*-value	ηG^2
Theta	3.03 (1, 22)	.096	.01	33.94 (2.35, 51.60)	<.001***	.15	.12 (1, 22)	.730	.01	<.01 (1, 22)	.957	<.01	.23 (2.35, 51.60)	.828	<.01	6.19 (1.75, 38.47)	.006**	.02	1.27 (1.75, 38.47)	.290	<.01
Alpha	7.69 (1, 22)	.011*	.01	22.54 (1.49, 32.85)	<.001***	.10	.20 (1, 22)	.661	<.01	1.36 (1, 22)	.257	<.01	.28 (1.49, 32.85)	.696	<.01	6.41 (2.05, 45.00)	.003**	.01	1.52 (2.05, 45.00)	.230	<.01
Beta	1.47 (1, 22)	.238	<.01	5.72 (1.36, 29.81)	.016*[1]	.04	.33 (1, 22)	.572	.02	.80 (1, 22)	.382	<.01	.43 (1.36, 29.81)	.577	<.01	1.63 (2.05, 45.07)	.207	<.01	1.09 (2.05, 45.07)	·.347	<.01

Table S-F.3 - Post-hoc pairwise comparisons of interaction effects between stimulation side and regions of interest (ROIs) on electroencephalography (EEG) theta and alpha power during painful cold pressor task (CPT) stimulation across all participants. All variables were square-root transformed before performing these analyses. The means and standard errors (SE) are post-hoc estimations based on the variables included in the repeated-measures analysis of variance. Whether the left or right hemisphere were ipsi- or contralateral to the stimulation side differed across participants due to randomisation of stimulation side. The ROI electrodes refer to: frontal = Fp1, AF3, F7, F3, F4, F8, AF4, Fp2, Fz; central-temporal = FC1, FC5, T7, C3, C4, T8, FC6, FC2, Cz; parietal = CP1, CP5, P7, P3, Pz, P4, P8, CP6, CP2; occipital = PO3, O1, Oz, O2, PO4. * $p < .05$. ** $p < .01$. *** $p < .001$.

Frequency band	ROI	Mean (SE) contralateral	Mean (SE) ipsilateral	Mean difference	SE	*p*-value
Theta	Frontal	.70 (.03)	.70 (.03)	|.02|	.02	.177
	Central-temporal	.61 (.03)	.61 (.03)	0	.02	.988
	Parietal	.73 (.04)	.61 (.04)	|.12|	.04	.015*
	Occipital	.81 (.03)	.79 (.04)	|.02|	.02	.340
Alpha	Frontal	.56 (.04)	.57 (.03)	0	.01	.713
	Central-temporal	.56 (.04)	.51 (.04)	|.05|	.02	.041*
	Parietal	.68 (.06)	.56 (.05)	|.13|	.04	.005**
	Occipital	.74 (.06)	.72 (.06)	|.03|	.02	.270

Table S-F.4 - Post-hoc pairwise comparisons of regions of interest (ROIs) effects on beta power during painful cold pressor task (CPT) stimulation across all participants. All variables were square-root transformed before performing these analyses. The means and standard errors (SE) are post-hoc estimations based on the variables included in the repeated-measures analysis of variance. The ROI electrodes refer to: frontal = Fp1, AF3, F7, F3, F4, F8, AF4, Fp2, Fz; central-temporal = FC1, FC5, T7, C3, C4, T8, FC6, FC2, Cz; parietal = CP1, CP5, P7, P3, Pz, P4, P8, CP6, CP2; occipital = PO3, O1, Oz, O2, PO4. * $p < .05$. ** $p < .01$. *** $p < .001$.

ROI (*mean*, *SE*)	Frontal	Central-temporal	Parietal
	Mean difference (*SE*), *p*-value		
Frontal (.44, .03)	-		
Central-temporal (.39, .03)	|.06| (.02), .012*	-	
Parietal (.41, .03)	|.04| (.03), .936	|.02| (.02), 1.00	-
Occipital (.46, .04)	|.02| (.03), 1.00	|.08| (.02), .006**	|.06| (.01), <.001***
